# Concurrent Drought Stress and Vascular Pathogen Infection Induce Common and Distinct Transcriptomic Responses in Chickpea

**DOI:** 10.3389/fpls.2017.00333

**Published:** 2017-03-14

**Authors:** Ranjita Sinha, Aarti Gupta, Muthappa Senthil-Kumar

**Affiliations:** National Institute of Plant Genome ResearchNew Delhi, India

**Keywords:** *Ralstonia solanacearum*, microarray, unique response, cellulose and lignin biosynthesis, combined stress, drought-pathogen stress, molecular response

## Abstract

Chickpea (*Cicer arietinum*); the second largest legume grown worldwide is prone to drought and various pathogen infections. These drought and pathogen stresses often occur concurrently in the field conditions. However, the molecular events in response to that are largely unknown. The present study examines the transcriptome dynamics in chickpea plants exposed to a combination of water-deficit stress and *Ralstonia solanacearum* infection. *R. solanacearum* is a potential wilt disease causing pathogen in chickpea. Drought stressed chickpea plants were infected with this pathogen and the plants were allowed to experience progressive drought with 2 and 4 days of *R. solanacearum* infection called short duration stress (SD stresses) and long duration stress (LD stresses), respectively. Our study showed that *R. solanacearum* multiplication decreased under SD-combined stress compared to SD-pathogen but there was no significant change in LD-combined stress compared to LD-pathogen. The microarray analysis during these conditions showed that 821 and 1039 differentially expressed genes (DEGs) were unique to SD- and LD-combined stresses, respectively, when compared with individual stress conditions. Three and fifteen genes were common among all the SD-stress treatments and LD-stress treatments, respectively. Genes involved in secondary cell wall biosynthesis, alkaloid biosynthesis, defense related proteins, and osmo-protectants were up-regulated during combined stress. The expression of genes involved in lignin and cellulose biosynthesis were specifically up-regulated in SD-combined, LD-combined, and LD-pathogen stress. A close transcriptomic association of LD-pathogen stress with SD-combined stress was observed in this study which indicates that *R. solanacearum* infection also exerts drought stress along with pathogen stress thus mimics combined stress effect. Furthermore the expression profiling of candidate genes using real-time quantitative PCR validated the microarray data. The study showed that down-regulation of defense-related genes during LD-combined stress resulted in an increased bacterial multiplication as compared to SD-combined stress. Overall, our study highlights a sub-set of DEGs uniquely expressed in response to combined stress, which serve as potential candidates for further functional characterization to delineate the molecular response of the plant to concurrent drought-pathogen stress.

## Introduction

Chickpea (*Cicer arietinum*) is second largest cultivated legume crop in world. The crop is vulnerable to drought stress (Gaur et al., 2012) as well as to wilt diseases like Fusarium wilt caused by *Fusarium oxysporum* f. sp. *ciceris* (Nene et al., 2012). Under field conditions drought and pathogen stress often occurs simultaneously. Moreover, the combination of drought and pathogen stress has been noted to be devastating for growth and yield of crop plants (Olson et al., 1990; McElrone et al., 2001). These two stressors are noted for their influence on each other’s interaction with plant, which might result in either negative or positive impact on the plants. Several studies in Arabidopsis, bean, grapevine have shown that drought stress makes the plant vulnerable to pathogen infection (McElrone et al., 2001; Mayek-Péreza et al., 2002; Mohr and Cahill, 2003; Prasch and Sonnewald, 2013). Conversely, reports also indicate that drought stress enhances the defense response of plants against pathogen (Achuo et al., 2006; Ramegowda et al., 2013; Hatmi et al., 2015). Pathogen infection has also been shown to alter the response of plants to water-deficit conditions. For example, wilt causing pathogens inhabit the xylem tissues, resulting in vascular dysfunction, and consequently causes a drought-like effect in plants (Douglas and MacHardy, 1981; Genin, 2010).

Overall, it is observed that the water deprivation induced during combined occurrence of drought and vascular pathogen infection increases the susceptibility of plants against pathogen as well as drought stress (McElrone et al., 2001; Kavak and Boydak, 2011; Choi et al., 2013; Ochola et al., 2015). Therefore it is important to understand the impact of combined stress and the cognate defense strategies adopted by plants to circumvent the concurrent onslaught of drought and vascular pathogen. However, the studies to understand the underlying molecular responses to combined drought and vascular pathogen are limited (Choi et al., 2013). At this juncture, in our present study we tried to dissect the molecular response of chickpea under combined drought and *Ralstonia solanacearum* infection. *R. solanacearum* is one of the most devastating wilt causing vascular pathogen having a broad host range and known to invade more than 200 different plants species (Genin, 2010). *R. solanacearum* colonizes xylem tissue and secretes massive amount of exopolysaccharides, which subsequently interrupts xylem function and leads to the wilting of the plant (Genin, 2010). *R. solanacearum* infection in chickpea was observed to result in a mild to severe disease symptoms which include yellowing, wilting and cell death; however, it was also observed that *R. solanacearum* does not provoke sudden plant death in leaf-inoculated plants (Sinha et al., 2016). The molecular responses of plants against *R. solanacearum* infection has been extensively studied (Hwang et al., 2011; Ishihara et al., 2012; Chen et al., 2014; Prasath et al., 2014; Zuluaga et al., 2015); however, so far no attempt has been made to understand the transcriptomic responses of plant to combined drought and *R. solanacearum* infection.

Recently, Sinha et al. (2016) showed that drought restricts the multiplication of *R. solanacearum* in chickpea which suggests that combined stress can induce robust defense responses in chickpea, thus proposes the need to explore transcriptomic responses of chickpea under combined drought and *R. solanacearum* infection. Since *R. solanacearum* infection in chickpea causes disease symptoms after 6 days post infection (dpi) (Sinha et al., 2016), it has been hypothesized that *R. solanacearum* probably induces early and late defense responses at 2 and 4 dpi, respectively.

In the present study, transcriptomic responses of chickpea toward combined and individual *R. solanacearum* infection and drought stress was investigated using microarray at two time points namely short duration (SD) and long duration (LD). The transcriptomic changes induced during LD and SD combined stresses were categorized as unique (responses observed only during combined stress) and common (responses overlapping between individual and combined stresses) responses.

## Materials and Methods

### Plant Material and Growth Conditions

Chickpea plants (variety ICC 4958) were grown in pots (5 inches diameter and 5 inches height) containing 500 gm of 3:1 mixture (vol/vol) of air dried peat (Prakruthi Agri Coco peat Industries, Karnataka, India) and vermiculite (Keltech Energies Pvt Ltd., Maharashtra, India) in an environmentally controlled growth chamber (PGR15, Conviron, Winnipeg, MB, Canada) with diurnal cycle of 12-h-light/12-h-dark, 200 μEm^-2^s^-1^ photon flux intensity, 22°C temperature and 75% relative humidity. Pots were bottom irrigated every 2 days with half strength Hoagland’s medium (**TS1094**, Hi-media Laboratories, Mumbai, India).

### Inoculum Preparation for Pathogen Infection

*Ralstonia solanacearum* procured from Indian Type Culture Collection (ITCC# BI0001), IARI, New Delhi, India was grown in LB medium (without antibiotic) at 28°C to OD_600_ = 0.6 with a continuous shaking of 200 rpm for 2.5 h. Culture was pellet down by centrifuging at 3500 × *g* for 10 min, washed thrice with sterile distilled water, and re-suspended in sterile distilled water to OD_600_ = 0.005 corresponding to 7 × 10^5^ colony forming units (cfu)/ml.

### Stress Treatments

Pathogen stresses namely SD-pathogen (2 days post *R. solanacearum* infection) and LD-pathogen (4 days post *R. solanacearum* infection) and drought stress namely SD-drought, SD or fast drought imposition and LD-drought, LD or slow drought imposition were used for this study. The two drought methods were included with incite that duration of drought imposition determines the plant’s biochemical and molecular responses and therefore may affect the combined stress outcome. With SD- and LD-drought and SD- and LD-pathogen stresses, two combinations of combined stress treatments namely SD-combined (fast drought with 2 days of *R. solanacearum* infection) and LD-combined stress (slow drought with 4 days of *R. solanacearum* infection) were considered. Altogether, six stress treatments were SD-combined (SD drought stress with 2 days of *R. solanacearum* infection), SD-pathogen (2 days of *R. solanacearum* infection), SD-drought [SD drought stress, 35% field capacity (FC)], LD-combined (LD drought with 4 days of *R. solanacearum* infection), LD-pathogen (4 days of *R. solanacearum* infection) and LD-drought (30% FC). Four plants were maintained for each stress treatment, along with absolute control and mock control and were placed in growth chamber using completely randomized design (CRD).

Drought stress was imposed by withholding the water, and stress level was assessed by measuring pot mix using gravimetric method following Sinha et al. (2016). For SD- and LD-drought stress treatments, water was withheld for 10 and 15 days for 29- and 24-day-old plants to attain 35% (SD-drought) and 30% FC (LD-drought), respectively, on 39th day of plant growth. *R. solanacearum* inoculation was done by vacuum infiltration, in which the plants were upturned in a beaker placed in vacuum chamber containing *R. solanacearum* culture (7 × 10^5^ cfu/ml) with 0.02% Silwet L77 (Lehle seeds, Fisher Scientific, Waltham, MA, USA) and vacuum of 8.7 psi was applied for 10 min. Plants were briefly rinsed instantly after infiltration. To avoid the entry of bacterial suspension into the pot mix, the pot surface was covered with polythene wrap prior to infiltration. *R. solanacearum* was infiltrated into 35- and 37-day-old plants and samples were collected after 4 and 2 days of infiltration for LD- and SD-pathogen stress treatments, respectively. In case of LD- and SD-combined stress treatments, water was withheld for 8 and 11 days for 29- and 24-day-old plants to attain 40 and 35% FC, respectively. Following this, plants were vacuum infiltrated with 7 × 10^5^ cfu/ml of *R. solanacearum* culture and were allowed for 2 and 4 days of progressive drought stress post *R. solanacearum* infection. After 2 and 4 days of progressive drought stress, FC of SD- and LD-combined stressed plants were 35 and 30%, respectively. Since plants at 60% FC showed better growth than 100% FC because of the high water holding capacity of pot mix, absolute and mock controls were maintained at 60% FC. For mock control, plants were infiltrated with water containing 0.02% Silwet L77. Leaf samples for all the treatment were collected from 39 days old plant for microarray analysis. The methodology of stress imposition is diagrammatically illustrated in Supplementary Figure S2.

### Assessment of *in planta* Bacterial Numbers

Total *in planta* colony forming units (cfu) of *R. solanacearum* were counted at 0 and 2 days post treatment (dpt) for SD-pathogen and SD-combined stress, and at 0, 2, and 4 dpt for LD-pathogen and LD-combined stress experiments. The leaflets harvested from infiltrated plants were weighed and surface sterilized with 0.01% H_2_O_2_ for 5 s and subsequently homogenized in 100 μl of sterile water. After serial dilution in sterile water, leaflet homogenate was plated on LB medium without antibiotics. Since there was no significant difference in dry weight (DW) as well as fresh weight (FW) ratio for individual pathogen and combined stressed plants, FW was accounted in calculating bacterial numbers. Bacterial count was expressed as log transformed values of cfu/mg FW of leaflet.

Cfu/mg was calculated using the following formula:

$$\begin{array}{c} CFU/mg\;=\;\frac{\frac{\mathrm{Number}\mathrm{of}\mathrm{colonies}\times \mathrm{volume}\mathrm{of}\mathrm{homogenate}\;(\mathrm{ml})\times \mathrm{dilution}\mathrm{factor}}{\mathrm{volume}\mathrm{plated}\;(\mathrm{ml})}}{\mathrm{weight}\mathrm{of}\mathrm{the}\mathrm{leaflet}(\mathrm{mg})} \end{array}$$

### Relative Water Content

For all the six stress treatments and controls, relative water content (RWC) was measured at the end of the experiment (39-day-old plant). Moreover, RWC was also measured for SD- and LD-drought at 8 and 11 days after start of drought imposition, respectively. To measure the RWC, FW of leaflet samples was measured immediately after sample collection and samples were hydrated by floating on de-ionized water. Turgid weight (TW) was noted once leaflets attended full turgidity after 6 h at 22°C temperature. Samples were then oven dried at 60°C until they reach constant weight after 3 days, and DW was measured. RWC was calculated using the formula:

$$\begin{array}{c} \mathrm{RWC}(\%)\;=\;\frac{\mathrm{FW}-\mathrm{DW}}{\mathrm{TW}-\mathrm{DW}}*100 \end{array}$$

### Microarray Analysis

Microarray analysis for all treatments was performed in two biological replicates after taking clue from previous studies with two biological replicates (Yang et al., 2012; Liu et al., 2014; Chan et al., 2016). Leaf samples from two plants were pooled to make one biological replicate following recommendation by Kendziorski et al. (2005). The pooled leaves were mixed properly and a part of it was used for RNA isolation. Customized chickpea microarray chip with 60 mer oligonucleotide probe (Agilent_AMADID – 037094, Agilent technologies, Palo Alto, CA, USA) was used for the study. Entire experiment was performed once. The sampling method is thoroughly described in Supplementary Figure S3 and a flow chart describing the steps in the microarray analysis is shown in Supplementary Figure S4.

#### RNA Isolation

Total RNA from leaf tissue for all the treatments and controls was isolated using TRIzol reagent (Cat# 15596026, Invitrogen, Carlsbad, CA, USA) following manufacturers’ protocol. Further, RNA was purified using RNeasy minikit (Cat# 74104, Qiagen, Hilden, Germany) and quantified using NanoDrop ND-1000 spectrophotometer (Thermo Scientific, Waltham, MA, USA). Quality of RNA was assessed on a Bioanalyzer (Agilent technologies, Palo Alto, CA, USA).

#### Labeling

Samples for transcriptome analysis were labeled using Agilent Quick-Amp labeling Kit (Cat# 5190-0442, Agilent Technologies, Palo Alto, CA, USA). Total RNA (RIN numbers 6–8, 500 ng) was reverse transcribed at 40°C using oligo(dT) primer tagged to a T7 polymerase promoter and converted to double stranded cDNA to be used as template for cRNA generation. cRNA was generated by *in vitro* transcription and the dye Cy3 CTP was incorporated during this step. The cDNA synthesis and *in vitro* transcription procedures were carried out at 40°C. Labeled cRNA was purified using Qiagen RNeasy columns (Cat# 74106, Qiagen, Hilden, Germany) and quality was assessed for yields and quality using the Nanodrop ND-1000.

#### Hybridization and Scanning

Labeled cRNA (600 ng) was fragmented at 60°C and hybridized on to a Chickpea GXP_8X60K (AMADID: 037094) microarray chip. Fragmentation of labeled cRNA and hybridization were done using Gene Expression Hybridization Kit (Cat# 5190-0404, Agilent Technologies, California, USA). Hybridization was carried out in SureHyb Microarray Hybridization Chamber (Cat# G2534A, Agilent Technologies, Palo Alto, CA, USA) at 65°C for 16 h. The hybridized slides were washed with Agilent Gene Expression Wash Buffers (Cat# 5188-5327, Agilent technologies, Palo Alto, CA, USA) and scanned using the Agilent microarray scanner (Model# G2600D, Agilent Technologies, Palo Alto, CA, USA).

#### Data Analysis

Scanned images were quantified using Feature Extraction Software (Version-11.5 Agilent technologies, Palo Alto, CA, USA). Feature extracted raw data was analyzed using GeneSpring GX software (Version 12.1, Agilent technologies, Palo Alto, CA, USA). Normalization of the data was done in GeneSpring GX using the 50th percentile shift (where *n* has a range from 0 to 100 and *n* = 50 is the median) and differential expression patterns were identified for each sample. Fold expression values for combined stresses and pathogen stresses were obtained with respect to mock control and for drought stresses were obtained with respect to absolute control samples. Statistical unpaired student’s *t*-test was applied among the replicates and *p*-value was calculated based on volcano plot algorithm (GeneSpring GX, Agilent technologies, Palo Alto, CA, USA). Microarray dataset was submitted to Gene Expression Omnibus (**GSE89228**). Differentially expressed genes (DEGs) with *p*-value < 0.05 were selected. Different fold change cut off values ranging from 0.5 to 1.5 were tried with intension of including genes with high fold change expression. Also we looked for the fold change value which did not eliminate genes related to hormone, biotic stress, abiotic stress and xylem modification. Consequently, up-regulated genes with fold > 1 (log base2) and down-regulated genes with fold > -1 (log base2) were selected for further studies.

### Annotation of Transcripts and *in silico* Expression Profiling

Gene annotation data was retrieved from chickpea transcriptome database^1^ (Verma et al., 2015). Further, annotation of some of the genes was updated by performing BLASTN search against chickpea database (taxid: 3827) available in NCBI. Differentially regulated genes were clustered using hierarchical clustering based on Pearson coefficient correlation algorithm^2^. Venn diagrams were generated to view the common and unique genes in various conditions (Venny 2.0^3^). Orthologous genes of *Arabidopsis thaliana* were obtained from chickpea transcriptome database^4^ (Verma et al., 2015) and their GO enrichment was performed using AgriGO singular enrichment analysis^5^ with default setting of Arabidopsis gene model (TAIR10) background and other parameters (statistical test method: Fisher; multi-test adjustment method: Yekutieli FDR under dependency; significance level: 0.05^5^). Finally, heat maps were generated using GENE-E software^6^.

### Real-Time PCR Analysis

RNA from leaf tissue (100 mg FW) was isolated by TRIzol reagent (Cat# 15596018, Thermo Fisher Scientific, Waltham, MA, USA) following manufacturer’s guidelines. Quality, quantity and integrity of RNA were verified by agarose gel electrophoresis and NanoDrop (Thermo Scientific, Waltham, MA, USA). The RNA samples with O.D. ratios at 260/280 nm in the range of 1.9–2.1, and at 260/230 nm in the range of 2.0–2.3 were used for RT-qPCR. First strand cDNA was synthesized from 5 μg of DNase treated total RNA in a reaction volume of 50 μl using Verso cDNA synthesis kit (Cat# K1621, Thermo Fisher Scientific, Waltham, MA, USA). Primers were obtained from Sigma–Aldrich, St. Louis, MO, USA. Details of gene-specific primers used for the RT-qPCR are provided in Supplementary Table S1. Reaction mix was prepared by adding 1 μl of two fold-diluted cDNA and 1 μl of each of the specific primers (10 μM/μl) to 5 μl of SYBR Green PCR master mix (Cat# 4309155, Thermo Fisher Scientific, Waltham, MA, USA) and final volume was made to 10 μl. The reaction was run in ABI Prism 7000 sequence detection system (Applied Biosystems, Foster City, CA, USA). Relative fold change in gene expression was quantified using 2^-ΔΔCt^ method (Livak and Schmittgen, 2001) using *CaActin1* (EU529707.1) as endogenous control to normalize the data. For all the RT-qPCR experiments, three independent biological replicates and two technical replicates were included. For statistical analysis, the relative quantification value (RQ) was transformed to log_2_ value and test of significance was performed by one sample *t*-test.

## Results

### Differential Transcriptomic Response of Chickpea under Combined and Individual Drought and *Ralstonia solanacearum* Stresses

To understand the transcriptomic responses of chickpea to combined drought and vascular pathogen *Ralstonia solanacearum*, combined stress experiments were conducted by imposing two levels of drought and pathogen stress. The study was conducted with two types of drought stress namely SD drought or fast drought (SD-drought) and LD drought or slow drought (LD-drought) with the premise that duration and severity of drought stress determines the plants biochemical and molecular responses and therefore may differentially influence the combined stress response in chickpea. Two time points in *R. solanacearum* growth, i.e., 2 days post infiltration (dpi) (SD-pathogen stress) and 4 dpi (LD-pathogen stress) were selected for the evaluation of transcriptomic response of chickpea against *R. solanacearum*. With the two types of drought stress and two time points of *R. solanacearum* multiplication, we imposed two types of combined stress namely SD (SD-combined stress, fast drought with 2 days post combined stress treatment, dpt) and LD (LD-combined stress, slow drought with 4 dpt) (Supplementary Figure S2A). Before initiating the combined stress study, *R. solanacearum* was first assessed for its pathogenicity in chickpea. It was found that at an infiltrated concentration of 7 × 10^5^ cfu/ml, *R. solanacearum* was multiplying *in planta* and caused disease symptoms varying from mild water soaked lesions and yellowing to severe cell death and wilting. Also, bacterial ooze was noticed from base of the leaflet (Supplementary Figure S1). In the present study, drought stress was observed to induce reduction in leaf RWC. LD-drought stress showed more reduction in leaf water, i.e., 64% RWC after 11 days of water-withholding compared to SD-drought stress after 8 days of water withholding, i.e., 75% RWC (Supplementary Figure S2D) indicating an increased severity of drought stress with increased drought duration. Plants exposed to SD drought (FC-35%) and LD drought (FC-30%) showed 73 and 52% RWC, respectively, compared to 86% RWC in control plants after 10 and 15 days of drought treatment, respectively. *R. solanacearum* infiltration resulted in water soaking leading to 86% RWC in SD-combined stressed and 87% RWC in LD-combined stressed plants which were close to those of SD-pathogen (86%) and LD-pathogen (83%) stressed plants after 2 and 4 days post combined stress treatment (Supplementary Figure S2E). Notable reduction *in planta* multiplication of *R. solanacearum* was observed under SD-combined stress compared to SD-pathogen stress (Supplementary Figure S2B). However, *in planta* bacterial count was unchanged under LD-combined stress compared to LD-pathogen stress treatments (Supplementary Figure S2C).

Transcriptomic alterations in chickpea plants challenged with combined drought and *R. solanacearum* stress (SD- and LD-combined) and individual drought (SD- and LD-drought) and *R. solanacearum* stress (SD- and LD-pathogen) were studied by microarray analysis. Microarray data was submitted to Gene Expression Omnibus (GEO# GSE89228). The DEGs (Log_2_ FC ≥± 1) under drought stress compared to absolute control, and under combined and pathogen stresses compared to mock control were screened using unpaired *t*-test (*p* ≤ 0.05). Putative annotation of DEGs were obtained from chickpea transcriptome database^7^ (Verma et al., 2015) and also by homolog search using nucleotide BLAST^8^ against chickpea (NCBI taxid: 3827) database. The DEGs for which putative annotation could not be obtained by BLASTN or BLASTX were termed as unannotated DEGs (Supplementary Table S2). Under both SD-pathogen and LD-pathogen stress treatments, many genes involved in defense response (*WRKY33, MAP KINASE 11*, and *DEFENSIN*) were up-regulated. Similarly, under SD-drought and LD-drought stress treatments, genes involved in signaling, biosynthesis of abscisic acid (ABA) and osmo-protectants namely the genes encoding for LATE EMBRYOGENESIS ABUNDANT 5 (LEA5), LOW-TEMPERATURE-INDUCED 65 KDA PROTEIN were up-regulated. Concordant with these observations, genes involved in both defense responses and abiotic stress tolerance (genes encoding for LEA and RESPIRATORY BURST OXIDASE HOMOLOG B) were differentially expressed in SD-combined and LD-combined stresses, which conforms to the nature of the stressors (Supplementary Figure S5 and File S1). The majority of top most up-regulated genes were belonging to stress responsive and cell wall modification categories under combined stress (Supplementary Figure S5 and File S1).

**FIGURE 1:**
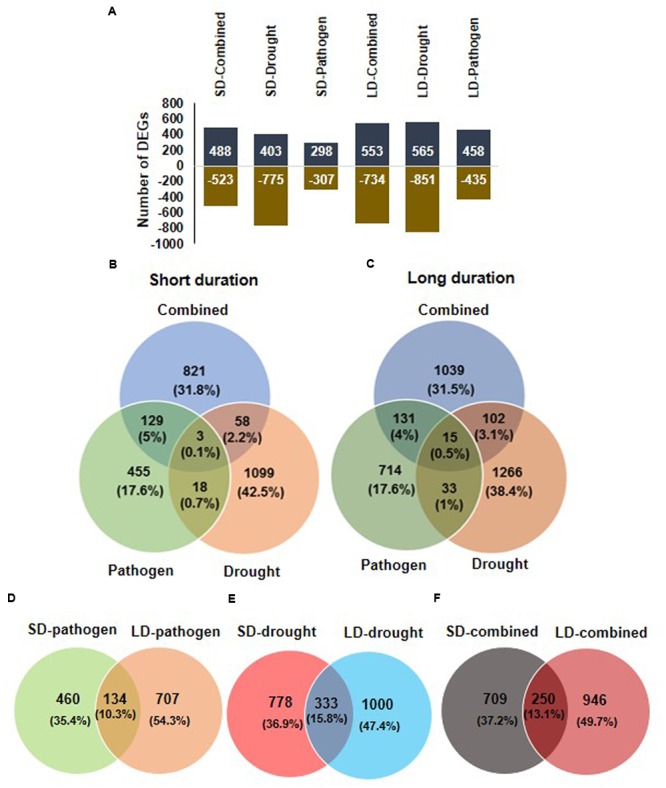
**Overview of differentially expressed genes (DEGs) in chickpea transcriptome in response to combined and individual stresses.** To study the transcriptomic changes in chickpea ICC4958 in response to pathogen (*Ralstonia solanacearum*), drought and combination of *R. solanacearum* and drought stress, chickpea plants were imposed with drought alone, pathogen alone and combined stress for short duration (SD; 2 days) and long duration (LD; 4 days) and named as SD-stresses and LD-stresses, respectively. DEGs over control in drought only treated plant and over mock in pathogen treated and combined stresses plants were obtained by microarray analysis. Total number of DEGs having a minimum fold change of 1 (Log_2_ transformed) and *p* < 0.05 under all six stress treatments, SD stresses (SD-pathogen, SD-drought, SD-combined) and LD stresses (LD-pathogen, LD-drought, LD-combined) are represented in graph **(A)**. Positive values in chart shows number of up-regulated and negative values represents number of down-regulated DEGs. The number of common and unique genes among different SD stresses and LD stresses are shown in Venn diagram **(B,C)**, respectively. Number of DEGs unique and common in SD- and LD-pathogen **(D)**, SD- and LD-drought **(E)** and SD- and LD-combined stress **(F)** are shown.

The number of up- and down-regulated genes under each stress condition is shown in **Figure 1**. The maximum numbers of DEGs were found in LD drought stress (1426 genes). Comparison of DEGs among SD stress treatments showed 821 genes (31.8%) out of 1011 genes to be uniquely up-regulated in response to SD-combined stress whereas, SD-combined stress treatment had 129 and 58 DEGs in common with the SD-pathogen and SD-drought stress, respectively (**Figure 1B**). Similarly, 1039 genes (31.5%) out of 1287 total DEGs were uniquely expressed under LD combined stress (**Figure 1C**). LD-combined stress and LD-drought stress had 102 DEGs in common, and both LD-combined and LD-pathogen had 131 genes in common (**Figure 1C**). The overlapping (common) and unique genes were also examined between SD-combined and LD-combined; SD-drought and LD-drought; SD-combined and LD-combined stress transcripts (**Figures 1D–F**). As a result a substantial variation in stress responsive transcriptome under SD-and LD-stresses was observed. The number of DEGs was more in LD stresses over SD stresses. For instance, number of DEGs under LD-pathogen was 841 compared to 594 under SD-pathogen stress treatment. Similarly, LD-drought stress treatment exhibited 1333 DEGs against 1111 DEGs under SD-drought stress. The LD-combined stress resulted in differential expression of 1196 genes over 959 DEGs under SD-combined stress. Each stress transcriptome had more number of unique DEGs and less number of common DEGs. LD-pathogen, LD-drought and LD-combined stress had 707, 1000, and 946 unique DEGs, respectively, as compared to 460, 778, and 709 unique DEGs in response to SD-pathogen, SD-drought and SD-combined stress, respectively. Moreover, very small percentages (10–15%) of genes were common between the respective SD and LD treatments (**Figures 1D–F**).

**FIGURE 2:**
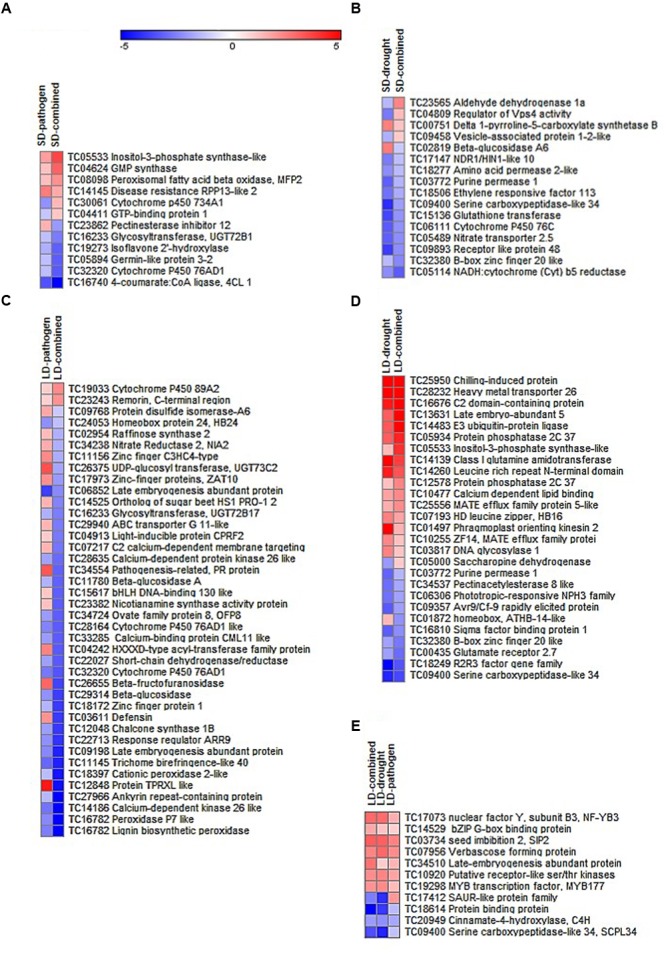
**Expression profile of DEGs common between combined and individual stresses.** The DEGs with more than one fold expression and *p* < 0.05 under SD treatments (SD-pathogen, SD-drought, SD-combined stress) and LD treatments (LD-pathogen, LD-drought, LD-combined) were compared and DEGs common between combined and individual stresses were selected. The heat maps represent DEGs which are shared between combined and individual stresses but have at least one fold difference in their expression between two treatments. Heat map **(A)** represents 12 shared genes with differential expression out of total 129 common genes between SD-pathogen and SD-combined and **(B)** represents 16 out of total 58 shared genes between SD-drought and SD-combined stress with different expression. Similarly, heat map **(C)** represents 40 genes with differential expression out of total 131 common genes between LD-pathogen and LD-combined and **(D)** represents 27 out of total 102 common genes between LD-drought and LD-combined stress. Expression level of DEGs common among all LD stresses are represented in heat map **(E)**. Color scale shows gene expression range where color bar in red and blue represents up- and down-regulated genes, respectively. Details of the genes shown in heat maps are available in Supplementary File S5.

The overlapping genes showed differential expression under different stresses. A few common genes between LD combined and LD drought stress (genes encoding for LEA5, E3 UBIQUITIN-PROTEIN LIGASE, PP2C37, INOSITOL 3 PHOSPHATASE SYNTHASE LIKE, and MATE EFFULUX FAMILY PROTEIN 5) had higher expression in LD-combined stress as compared to LD-drought stress. On the other hand, genes encoding for PATHOGENESIS RELATED (PR), DEFENSIN, PROTEIN TRRXL LIKE, and UDP-GLYCOSYLTRANSFERASE 73C2 (UGT73C2) proteins showed down-regulation in LD-combined stress as compared to their up-regulation in LD-pathogen stress. However, some of the down-regulated genes encoding for proteins in SD-pathogen stress like CYTOCHROME P450 734A1 (CYP734A1) and GTP BINDING PROTEIN 1 were induced in SD-combined stress (**Figure 2**). All the LD stress treatments (combined and individual) had 15 genes in common where four genes exhibited similar expression and 11 others exhibited tailored expression (**Figures 1B**, **2** and Supplementary File S5).

**FIGURE 3:**
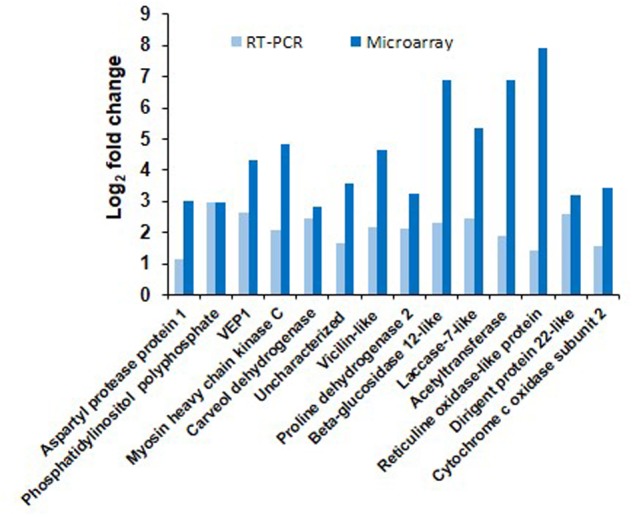
**Validation of microarray results by RT-qPCR.** Transcript expression pattern of selected genes from microarray data was validated by RT-qPCR. Bar chart represents fold change expression (Log_2_ FC) of the genes by RT-qPCR (light blue) and microarray (dark blue) under SD-combined stress. CaActin was used as reference gene for the qPCR normalization. Fold change was calculated over mock control. Each bar represents average of two biological and two technical replicates. Results presented are from one experiment.

The results obtained from microarray were verified by real time qPCR analysis of 14 genes, which were up-regulated in SD-combined stress. The differential expression of genes noted from qPCR and microarray are represented in **Figure 3** for the comparison. We also checked the expression of these 14 genes in other stress treatments to determine their relevance under other combined or individual stress treatments. We observed up-regulation of genes encoding CYTOCHROME C OXIDASE and RETICULIN OXIDASE LIKE under SD-combined, LD-combined and LD-pathogen stresses. Similarly, genes encoding CARVEOL DEHYDROGENASE and VICILLIN LIKE had a very high up-regulation under SD-combined, LD-combined and LD-pathogen stresses when compared to SD and LD drought treatments. BETA-GLUCOSIDASE 12 LIKE, ACETYLTRANSFERASE, PROLINE DEHYDROGENASE 2, and ASPARTYL PROTEASE 1 encoding genes exhibited high up-regulation in both SD and LD combined stresses. DIRIGENT PROTEIN 22 and LACCASE 7 LIKE encoding genes had high expression under SD-combined, SD-pathogen and LD-pathogen stresses. Gene for VEIN PATTERNING 1 (VEP1) was equally up-regulated in all treatments (Supplementary Figure S6). With the qPCR results we show that except *VEP1*, all other genes selected from SD-combined dataset were either specifically expressed or had higher expression under SD-combined stress, LD-combined stress and LD-pathogen stress treatments as compared to rest of the treatments.

**FIGURE 4:**
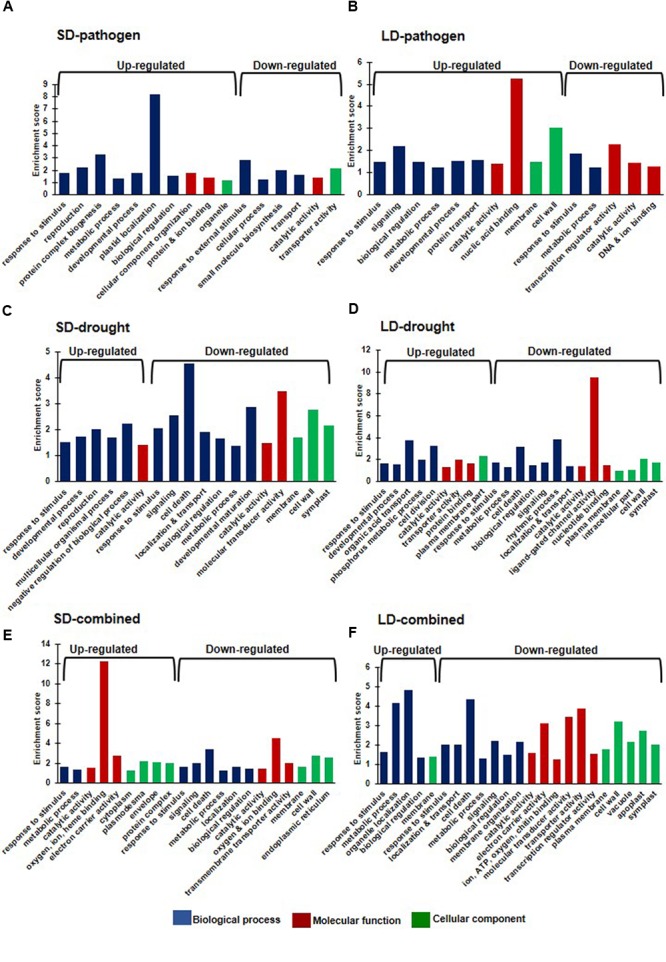
**Gene ontology (GO) enrichment analysis of individual and combined stress transcriptomes.** The *Arabidopsis thaliana* orthologs of chickpea genes were used for GO enrichment. *A. thaliana* orthologs were obtained for chickpea DEGs with more than one fold (Log_2_ converted) and *p* < 0.05 under six stress treatments (Supplementary Figure S2) from Chickpea Transcriptome Database (http://www.nipgr.res.in/ctdb.html). GO enrichment was done using AgriGO singular enrichment analysis with default settings (http://bioinfo.cau.edu.cn/agriGO/analysis.php). The enriched broad GO terms under each stress category are represented as bar diagram. Enrichment score of the GO terms are based on ratio of sample frequency and background frequency. Blue, red, and green color represents GO under biological process, molecular function and cellular component, respectively. Graphs show GO enrichment of DEGs under SD-pathogen **(A)**, SD-drought **(B)** SD-combined stress **(C)**, LD-pathogen **(D)**, LD-drought **(E)**, and LD-combined **(F)** stress categories. Number of *A. thaliana* orthologs obtained against total DEGs in each treatment is mentioned in Supplementary Table S2.

We further categorized the DEGs under each stress treatments based on gene ontology (GO) using Arabidopsis orthologs of chickpea genes (Supplementary Table S3). Overall, we found enrichment of up-regulated DEGs (from all stress treatments) in GO biological processes like response to stimulus, biological regulation, metabolic process, developmental process, transport, signaling, reproduction, cell death, cell division, negative regulation of biological process. Up-regulated DEGs were enriched in GO molecular functions like catalytic activity, protein, ion and nucleic acid binding, and cellular components like membrane, cell wall and symplast (**Figure 4**). Furthermore, SD- and LD-pathogen stressed transcriptomes exhibited up-regulated DEGs belonging to specific categories like ‘response to wounding/chitin/salt stress’ and ‘response to jasmonic acid (JA) and ABA’, while up-regulated DEGs under LD-pathogen stress were also enriched under ‘respiratory burst during defense response’ and ‘response to water deprivation’ category (Supplementary Figure S7). These results indicate that *R. solanacearum* infected plants manifest pathogen mediated drought stress like symptoms and oxidative burst to combat the pathogen at high titer. The down-regulated DEGs under SD- and LD- pathogen stress showed enrichment in categories like carbohydrate transmembrane transporter activity, fatty acid biosynthetic process, coumarin biosynthetic process, SA mediated signaling, negative regulation of defense response, systemic acquired resistance (SAR) and regulation of meristem growth (Supplementary Figure S7). Up-regulated DEGs under SD- and LD-drought stress were enriched in categories like response to water deprivation, ABA stimulus, negative regulation of biological process, cellular polysaccharide biosynthetic process whereas LD-drought treatment also showed enrichment in plasma membrane part, sugar and secondary active transporter activity and response to other organism categories. The down-regulated genes under SD- and LD-drought were enriched with GO processes: such as plant hypersensitive response, response to biotic stimulus, SA biosynthetic process, negative regulation of PCD, negative regulation of defense response, SAR, ligand gated ion channel activity and positive regulation of flavonoid biosynthetic process (Supplementary Figure S7).

The up-regulated DEGs under SD-combined stress genes were enriched under GO categories: mitochondrial membrane part, phosphorylation, response to temperature stimulus and oxidative stress, oxidation of organic compound and glutamine family amino acid metabolic process, while up-regulated DEGs under LD-combined stress, were enriched in secondary cell wall (SCW) biogenesis, response to water deprivation, ABA stimulus, lipid metabolic process regulation, hyperosmotic salinity response, hormone mediated signaling, glucosinolate biosynthesis and defense response GO categories. Down-regulated genes under SD- and LD-combined stress were majorly enriched in inorganic anion transmembrane transporter activity, protein serine/threonine kinase activity, negative regulation of cell death, MAPKKK cascade, regulation of hypersensitive response, defense response to fungus, salicylic acid (SA) biosynthetic process, SAR and positive regulator of flavonoid biosynthetic process (Supplementary Figure S7).

### Combined Stress and *R. solanacearum* Induces Transcriptome Changes Involved in Osmo-Protectant Accumulation

The genes involved in osmo-protectant biosynthesis, like those genes encoding for LEA, RAFFINOSE SYNTHASE, STACHYOSE SYNTHASE, VERBASCOSE SYNTHASE, DELTA 1-PYRROLINE-5-CARBOXYLATE SYNTHASE (P5CS), and PROLINE OXIDASE (involved in proline catabolism) were up-regulated in SD and LD-drought and SD and LD combined stress treatments. Moreover, the genes encoding RAFFINOSE BIOSYNTHESIS and LEA were also up-regulated in LD-pathogen stress indicating *R. solanacearum* mediated drought stress in the chickpea plants. Therefore, *R. solanacearum* infection itself mimics a dual abiotic and biotic stress in chickpea (Supplementary Figure S8). Results on hierarchal clustering among different treatments (based on differential gene expression) revealed closeness of LD-pathogen with SD-combined stress (Supplementary Figure S9) substantiating that *R. solanacearum* infection exerts both drought and pathogen stress in chickpea.

### Combined Stress Differentially Induces Genes Involved in Xylem Differentiation and Cellulose and Lignin Deposition

**FIGURE 5:**
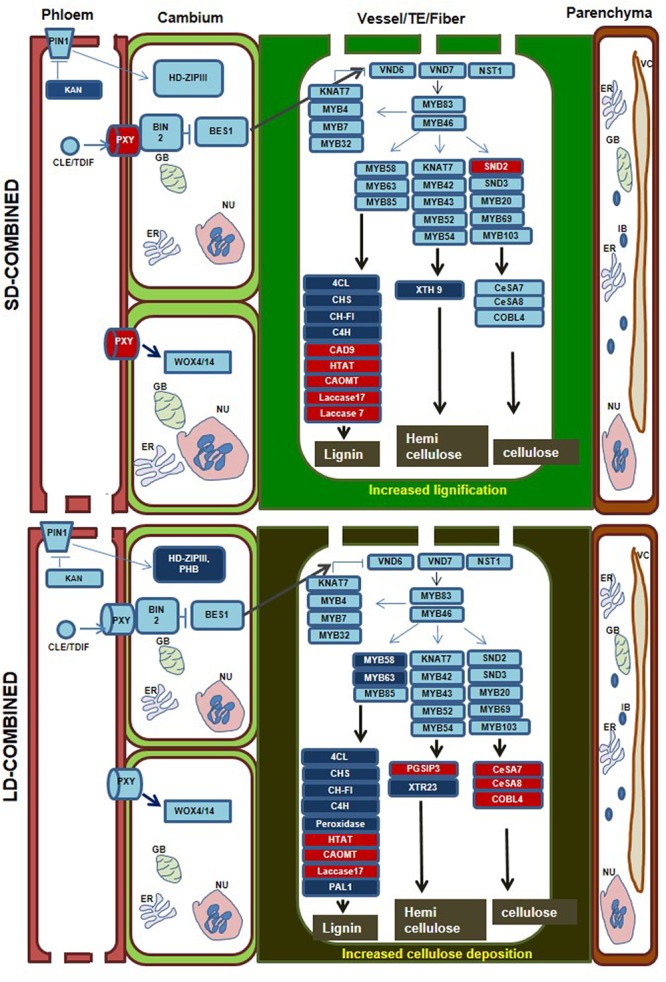
**Overview of differential expression of xylem differentiation and secondary cell wall (SCW) related genes in SD- and LD-combined stress treatments.** Diagrams represent vascular bundle structure with phloem, cambium and xylem vessel/fiber and xylem parenchyma. The order of the cell in the diagram is likely following the order present in dicot leaf where phloem is placed on abaxial followed by cambium and xylem on adaxial surface. DEGs with minimum fold change 1 (Log_2_ converted) were identified in each individual and combined stressed transcriptome over their respective controls. DEGs were mapped onto ‘xylem development and SCW deposition’ pathways based on literature information. Major molecular events related to xylem differentiation and SCW synthesis in xylem vessel, tracheary element and fiber are represented once in the same box. The SCW synthesis pathway is representing only those genes which are differentially expressed in the SD-combined and LD-combined stress transcriptome. Thickening of SCW in xylem vessel and TE are represented as thick green wall of the cell. Increased cellulose and lignin deposition in LD-combined stress is represented as dark thick green color wall. Genes in red boxes represents up-regulated expression and in dark blue boxes represents down-regulated expression. Genes in the boxes with the light blue color has no differential expression. Details of the genes in the boxes are mentioned in the Supplementary File S2.

Differentially expressed genes in each stress category were mapped onto pathways involved in xylem differentiation based on available literature information (Růžička et al., 2015; Rybel et al., 2016). During SD-combined stress, we observed down-regulation of the gene *KANADI* (one clade of the GARP family of transcription factors) that act as negative regulator of procambium/cambium and vascular tissue formation (Růžička et al., 2015; Rybel et al., 2016). *KANADI* also indirectly suppresses expression of HD-ZIPIII transcription factor which promotes meristem function and xylem tissue formation (Růžička et al., 2015; Rybel et al., 2016). Down-regulation of *KANADI* thus enhances the possibility of either cambial proliferation or xylem trans-differentiation in SD-combined stressed plants. Another gene encoding PHLOEM INTERCALATED WITH XYLEM (PXY) receptor which is responsible for the BRASSINAZOLE-RESISTANT 1 (BES) mediated signaling (Růžička et al., 2015; Rybel et al., 2016) was up-regulated in SD-combined stressed plants (**Figure 5**, Supplementary Figure S10 and File S2). This may result either in increased cambial proliferation or xylem differentiation. Moreover, the mapping of DEGs onto pathway for SCW synthesis during xylem formation revealed high and unique up-regulation of gene encoding for SECONDARY WALL-ASSOCIATED NAC DOMAIN 2 (SND2), a tier 3 gene regulator in cellulose synthesis (Růžička et al., 2015; Taylor-Teeples et al., 2015) under SD-combined stress. However, genes encoding CELLULOSE SYNTHASE 8 (CESA 8), COBRA LIKE 2, and COBRA LIKE 4 which are directly involved in cellulose biosynthesis (Mcfarlane et al., 2014) were not differentially expressed in SD-combined stress but were highly and uniquely up-regulated in LD-combined stress (**Figure 5**, Supplementary Figure S10). Among genes involved in lignin biosynthesis, the gene encoding for CINNAMYL-ALCOHOL DEHYDROGENASE 9 (CAD9) which catalyzes the terminal step of monolignol biosynthesis (Bonawitz and Chapple, 2010) showed high up-regulation under SD-combined and LD-pathogen stresses. Also, very high up-regulation of *LACCASE7* which is involved in polymerization of monolignol to lignin (Bonawitz and Chapple, 2010) was observed in SD-combined and LD-pathogen stress. Similarly, *LACCASE17* showed up-regulation in SD-combined, LD-combined and LD-pathogen stresses. However, the genes involved in earlier steps of phenylpropanoid pathway for monolignol biosynthesis were down-regulated in both SD-combined and LD-combined stress (**Figure 5**, Supplementary Figures S10, S11, and File S3). The genes acting at later stage or regulating lignin polymerization were up-regulated under SD-combined, LD-combined, and LD-pathogen stress. Therefore, we assume that genes involved in initial step of lignin synthesis (phenylpropanoid pathway) is up-regulated at early time point during combined stress for initiating lignin synthesis pathway and later may be down-regulated to maintain the metabolic load. This could possibly be because of feedback regulation mediated by high monolignol titer in the cell at 2 and 4 dpi. PAL which catalyzes first step in phenylpropanoid pathway for lignin biosynthesis has been reported to work under sophisticated regulatory control. Both Pal activity and *PAL* gene transcription are negatively regulated by *trans*-cinnamic acid (t-CA) (Zhang and Liu, 2015). A transient induction in *PAL* gene expression has been observed in many studies. In *A. thaliana*, the expression of *PAL1* and *CAD* genes upon *Pseudomonas syringae* pv. tomato DC3000 (Psd) infection had highest expression at 2 h post infection (hpi). However, *PAL* expression declined at 4 hpi and later time points, i.e., 8, 12, 24 hpi (Arabidopsis eFP Browser^9^). Schmelzer et al. (1989) reported a rapid and transient increase in *PAL* and *4CL* mRNA level from 4 to 8 hpi followed by rapid decline in Parsley leaves upon *Phytophthora megasperma* f. sp. *glycinea* (Pmg) infection. The transient increase followed by decline in PAL activity at 12 h post *R. solanacearum* infection in resistant and 18–30 h post *R. solanacearum* infection in susceptible tomato variety was also reported by (Vanitha and Umesha, 2009). Kubasek et al. (1992) suggested that flavonoid genes are sequentially induced in the order of the biosynthetic steps in the flavonoid pathway and this level of regulation may be achieved by feed-forward or feedback mechanisms utilizing phenylpropanoid intermediates themselves. These evidences indirectly support our argument of sequential and transient expression of genes involved in lignin biosynthesis.

Hemi-cellulose deposition in cell wall is another important step in xylem biosynthesis. We observed up-regulation of gene *GLYCOGENIN-LIKE STARCH INITIATION 3* (*PGSIP3*) also called GUX2 regulating hemi-cellulose biosynthesis (Mortimer et al., 2010) in only LD-pathogen stress. Whereas, the hemi-cellulose biosynthesis gene; *IRREGULAR XYLEM 15* (*IRX15*-like) (Brown et al., 2011) showed unique and high up-regulation in SD-combined stress only. Two hemicellulose biosynthetic genes encoding for PURVUS/GLZ1 and PECTIN METHYLESTERASE (PME) (Scheller and Ulvskov, 2010) were up-regulated during SD- and LD-drought stress, respectively (**Figure 5**, Supplementary Figures S10, S11 and Files S2, S3). This strongly suggests that combined stress results in induction of lignin biosynthesis and modification in xylem SCW.

Furthermore, we wanted to explore if up-regulation of genes involved in SCW formation is a general consensus under combined stress. Therefore we looked for the expression of genes encoding for CAFFEIC ACID O-METHYLTRANSFERASE (COAMT), LACCASE 7 and 17 and CESA7 and 8 genes in combined drought and Psd transcriptomic data from Gupta et al. (2016). We found absence of those genes in differentially expressed transcriptome of both drought first Psd later (DP) and Psd first and drought later (PD) treatments (Gupta et al., 2016) (Supplementary Figure S12). This indicates that combined drought and foliar pathogen stress may not employ SCW modification for combined stress tolerance.

### Combined Drought and *R. solanacearum* Stress Differentially Induces Phytohormone Biosynthesis and Signaling Genes

**FIGURE 6:**
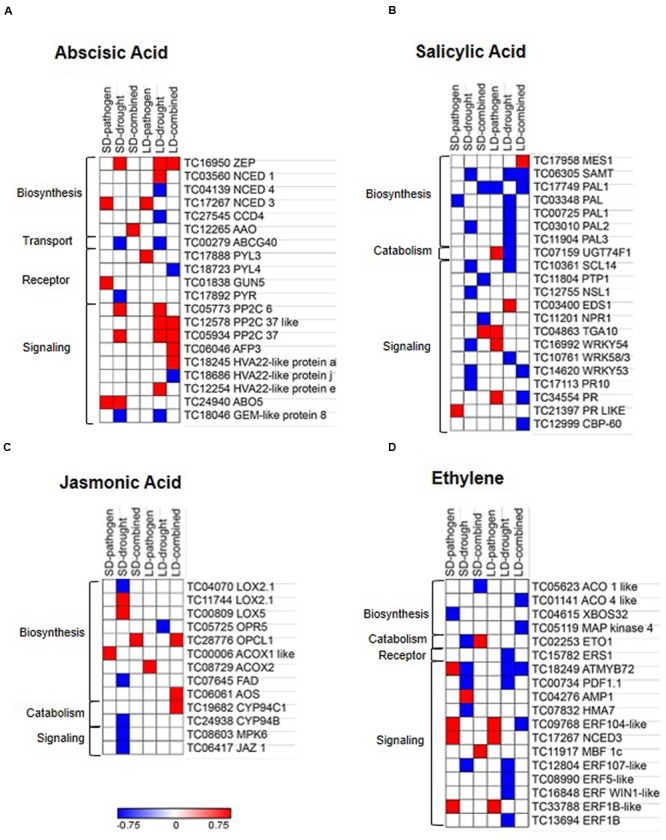
**Expression profile of hormone related genes in individual and combined stressed chickpea leaves.** DEGs with minimum fold change 1 (Log_2_ converted) were identified in each individual and combined stressed plants over their respective controls. DEGs were mapped onto ‘hormone related’ pathways using MAPMAN and KEGG softwares, literature and Arabidopsis hormone database (http://lifecenter.sgst.cn/orib/resourceDetail.do?resource.id=32682). Heat maps represent expression profile of hormone biosynthesis, catabolism, receptor, transport and signaling genes related to abscisic acid **(A)**, salicylic acid **(B)**, jasmonic acid **(C)**, and ethylene **(D)** under individual and combined stresses. Fold change values (over respective controls) were used to plot heat maps where color bar in red and blue represents up- and down-regulated genes, respectively, and white represents no differential expression. Details are mentioned in the Supplementary File S4.

The combined stress mediated alteration in hormone biosynthesis, catabolism, transport, and signaling were studied by mapping the DEGs onto hormone pathways based on available literature information. We found up-regulation of ABA biosynthesis genes in all stress treatments. We observed the stress treatment specific differential expression of ABA biosynthetic genes. ABA biosynthesis gene encoding 9-*CIS*-EPOXYCAROTENOID DIOXYGENASE 3 (NCED3) was specifically induced during SD- and LD-pathogen stress whereas during SD- and LD-drought stress, ABA biosynthetic genes encoding ZEAXANTHIN EPOXIDASE (ZEP) and NCED1 were induced. During SD-combined stress, ALDEHYDE OXIDASE (AAO) was specifically up-regulated, similarly in LD-combined stress only ZEP is found to be up-regulated. Down-regulation of gene encoding for ABA transporter ATP-BINDING CASSETTE G40 (ABCG40) was observed only during SD- and LD-drought. ABA receptor encoding genes were up-regulated in SD- and LD-pathogen stress and signaling genes *PP2C6* and *PP2C37* were up-regulated in SD-pathogen, SD-drought, LD-drought, and LD-combined stressed plants (**Figure 6A** and Supplementary File S4). PP2C is negative regulator of ABA signaling, however, has been shown to confer abiotic stress tolerance in many plants in ABA insensitive manner (Bhaskara et al., 2012; Zhang et al., 2013; Singh et al., 2015). Another negative regulator of ABA signaling geneABI5-interacting protein (*AFP3*) was also specifically up-regulated in LD-combined stress. The up-regulation of *PP2C* and *AFP3* under drought stress has been previously reported (Garcia et al., 2008) but its role under combined stress has not been seen yet. These results indicate that ABA signaling plays a major part in plant response under drought, *R. solanacearum* and combined stress.

Short duration- and long duration-pathogen stresses in chickpea induced up-regulation of SA and ethylene (ET) signaling genes encoding for TGACG (TGA) MOTIF-BINDING PROTEIN 10 (TGA10), PATHOGENESIS-RELATED GENE, WRKY54, ETHYLENE RESPONSE FACTOR104 LIKE (ERF104), and ERF1B (**Figures 6B,D** and Supplementary File S4). Contrastingly, we also encountered up-regulation of SA catabolism gene encoding for UDP-GLYCOSYLTRANSFERASE 74 F1 (UGT74F1) in LD-pathogen stress. SD- and LD-pathogen stress in chickpea also induced up-regulation of JA biosynthetic genes encoding ACYL-COA OXIDASE 1 and 2, respectively, but signaling genes were un-induced. SD-combined stress showed up-regulation of JA biosynthesis gene (*OPCL1*), down-regulation of SA repressor (*PROTEIN TYROSINE PHOSPHATASE1* (*PTP1*), Bartels et al., 2009) and up-regulation of SA and ET signaling genes *PR* and *MULTIPROTEIN BRIDGING FACTOR-1c (MBF1c)*, respectively (**Figure 6C** and Supplementary File S4). LD-combined stress treatment exhibited up-regulation of both JA biosynthetic genes *OPCL1, AOS*, and catabolism gene *CYTOCHROM P450 94C1* (**Figure 6C**). While, LD-combined stress had up-regulation of SA biosynthetic gene *MES1* (METHYLESTERASE 1), which converts methyl salicylate (MeSA) to SA (Dempsey et al., 2011), it showed down-regulation of SA signaling genes *WRKY53*, PR like, *CBP60* and ET signaling genes *MYB72* and *ERF104* (**Figures 6B,D**). Altogether, they indicate toward suppression of immunity in LD combined stress (Supplementary Figure S13C). Collectively, our results on transcriptome analysis of phytohormone related genes suggest an involvement of ABA, SA, and ET mediated signaling in modulating combined stress response in these plants.

Both SD- and LD-combined stress showed up-regulation of brassinosteroid (BR) inactivator *CYP734A1* (Vriet et al., 2013). Genes encoding for BR receptors BRASSINOSTEROID INSENSITIVE 1 (BRI1) and BRASSINOSTEROID INSENSITIVE 1 LIKE 2 (BRI1like 2) were up-regulated under LD-combined and SD-pathogen stresses, respectively (Supplementary Figure S13B). LD-combined stress also showed up-regulation of gibberellin (GA) biosynthesis gene GIBBERELLIN 3-BETA-DIOXYGENASE 1 (*GA1*), catabolism gene CYTOCHROME P450 714ALIKE (*CYP714A*like) and negative regulator of GA signaling gene; GIBBERELLIC ACID INSENSITIVE (*GAI*) indicating a loss of GA signaling in LD-combined stress (Supplementary Figure S13A). SD-combined stress resulted in up-regulation of genes encoding Auxin receptor; TOLL-INTERLEUKIN-RESISTANCE (TIR) and auxin transporter; ATP-BINDING CASSETTE B4 (ABCB4) (Supplementary Figures S13D and File S4).

### Differential Expression of Defense Related Genes in SD- and LD-Combined Stress Influences *R. solanacearum* Multiplication

**FIGURE 7:**
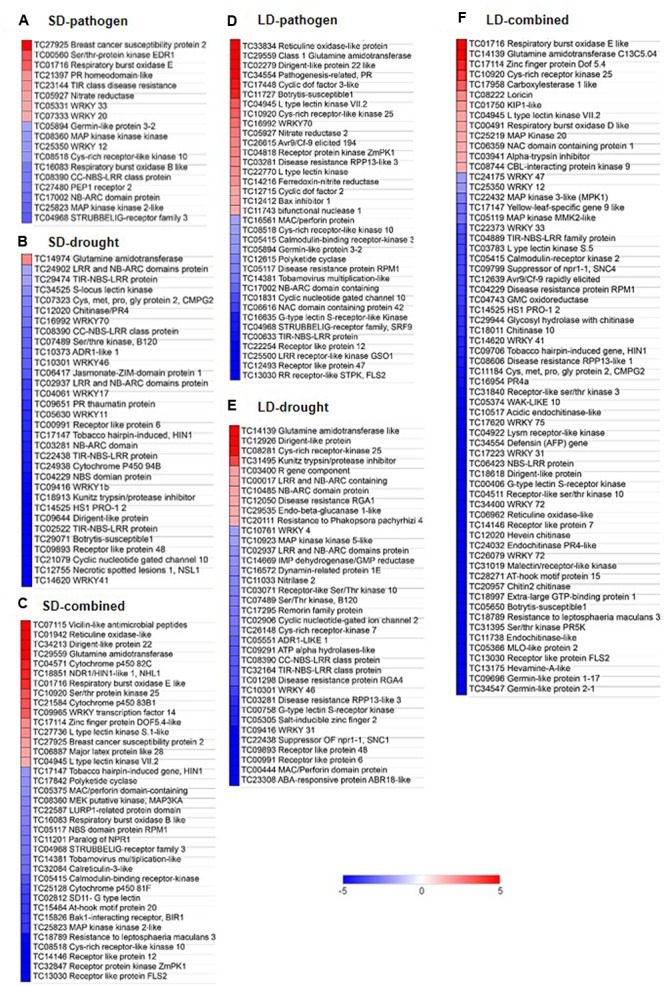
**Expression profile of genes involved in defense responses under individual and combined stress transcriptome.** DEGs with minimum fold change 1 (Log_2_ converted) were identified in each individual and combined stressed plants over their respective controls. DEGs involved in defense responses were identified using MAPMAN software and literature survey. Heat map represent expression profile of ‘defense response related’ genes under SD-pathogen **(A)**, SD-drought **(B)**, SD-combined **(C)**, LD-pathogen **(D)**, LD-drought **(E)**, and LD-combined stresses **(F)**. Fold change values (over respective controls) are represented in heat maps where color bar in red and blue represents up- and down-regulated genes, respectively.

We looked for the differential expression of biotic stress responsive genes in the all the six treatments. We found up-regulated expression of genes encoding PLEIOTROPIC DRUG RESISTANCE 3, ZINC FINGER PROTEIN, DOF ZINC FINGER PROTEIN DOF1.1, SER/THR-PROTEIN KINASE EDR1, (STPKEDR1) RESPIRATORY BURST OXIDASE HOMOLOG B (RBOHB), TIR CLASS DISEASE RESISTANCE, and NITRATE REDUCTASE under SD-pathogen stress. LD-pathogen stress showed up-regulation of defense related genes encoding RETICULIN OXIDASE LIKE PROTEIN, DISEASE RESISTANCE-RESPONSIVE (dirigent-like protein), GLUTAMINE AMIDO TRANSFERASE, PR, BOTRYTIS SUSCEPTIBLE 1, WRKY70, and RPP13. SD-combined stressed plants exhibited high up-regulation of genes encoding RETICULIN OXIDASE LIKE PROTEIN (also called BERBERINE BRIDGE ENZYME) involved in alkaloid biosynthesis, DISEASE RESISTANCE-RESPONSIVE (DIRIGENT-LIKE PROTEIN), LACCASE 7 involved in lignan and antioxidant synthesis to confer defense response in plant (**Figures 5**, **7**) (Dittrich and Kutchan, 1991; Zhao et al., 2013). SD-combined stress also exhibited high up-regulation of defense related genes encoding GLUTAMINE AMIDO TRANSFERASE, RBOHE like, STPK25, WRKY4, DOF5.4, and MAJOR LATEX PROTEIN LIKE 28 (MLP28) (**Figure 7**). SD-combined stressed transcriptome exhibited more number of up-regulated genes involved in defense response with high amplitude of expression as compared to SD-pathogen stress. This justifies the activated defense leading to decreased bacterial growth in SD-combined stress as compared to SD-pathogen. In LD-combined stress, we observed up-regulation of genes encoding for RBOHE like, GLUTAMINE AMIDOTRANSFERASE C13C5.04, ZINC FINGER PROTEIN DOF5.4, CYS-RICH RECEPTOR KINASE 25, however, several defense related genes such as genes encoding DEFENSIN, MLO LIKE PROTEIN, BOTRYTIS-SUSCEPTIBLE1, PR5, CHITINASE, RETICULINE OXIDASE-LIKE, DIRIGENT-LIKE PROTEIN, WRKY12/47/33/31/35/75, RESISTANCE TO LEPTOSPHAERIA MACULANS 3 (RLM3), TIR-NBS-LRR FAMILY PROTEIN, SUPPRESSOR OF NPR1-1 (SNC4), and DISEASE RESISTANCE PROTEIN RPM1 were down-regulated which otherwise were up-regulated under either SD-pathogen, LD-pathogen or SD-combined stress (**Figure 7**). Therefore, we conclude that the imposition of slow drought has a different impact on defense related transcriptome and disease resistance capacity of plant when compared to fast drought.

## Discussion

Xylem invading pathogens induce physiological drought stress in plants by blocking xylem and resultantly induce wilt (Genin, 2010). When wilt disease co-occurs with drought, plants are either resistant (Pennypacker et al., 1991; Sinha et al., 2016) or susceptible to the wilt pathogen (Abd El-Rahim et al., 1998; Choi et al., 2013). The combined occurrence of drought and vascular pathogen is often reported to reduce the plant height, total leaf area and decrease the hydraulic conductance, RWC and transpiration (Pennypacker et al., 1991; Abd El-Rahim et al., 1998; Choi et al., 2013). In our previous study (Sinha et al., 2016), the vascular pathogen *Ralstonia solanacearum* multiplication was shown to be significantly decreased after 6 days of infection under severe drought stress when compared to *R. solanacearum* infection alone in chickpea. In the present study, LD-combined stress did not change bacterial multiplication, however, SD-combined stress lead to decreased bacterial multiplication. The transcriptomic study under both SD- an LD-combined stresses unraveled responses unique to combined stress as well as responses common to both combined and individual stresses. Also SD- and LD-combined stress displayed very little overlap in transcriptomic responses between them which indicated that different durations of drought imposition in combined stress induces different transcriptomic changes in chickpea, consequently changing the overall effect on bacterial multiplication. Also, with increasing severity, the transcriptome complexity increased as reflected in more number of DEGs in LD-combined stress compared to SD-combined stress. Earlier, Gupta et al. (2016) reported increased resistance of *A. thaliana* to Psd under combined drought and Psd stress and they also reported that combined stress response differs with order of combined stress imposition (Gupta et al., 2016). However, Bidzinski et al. (2016) reported an increased susceptibility of rice plants toward *Magnaporthe oryzae* under intermittent drought and *M. oryzae* combined stress. Together these studies indicate that the plant’s response toward combined stress varies with the severity and order of stresses and the plant’s transcriptomic response also varies with continuous or intermittent drought stress. We also looked for the expression of certain unique genes from our study in transcriptomic data under combined drought and Psd (DPsd stress) stress in *A. thaliana* (Gupta et al., 2016) to compare if response to drought–foliar pathogen combination differs with drought–wilt pathogen combination. We could not find the differential expression of genes like LACCASE involved in lignin modification and flavonoid formation (Zhao et al., 2013) and CELLULOSE SYNTHASE involved in cellulose synthesis in DPsd transcriptome data. This suggests that unlike plant’s response toward combined drought and wilt pathogen, combined stress with drought and foliar pathogen does not involve SCW modification. Gupta et al. (2016) suggested the priming of basal defenses due to interaction of drought and pathogen derived responses in combined stressed plants as a contributory factor for the resistance response observed under combined stress.

In the present study, we observed that *R. solanacearum* infection induces expression of genes involved in SA and ET signaling, biotic stress response and cell wall modification in chickpea. Earlier *R. solanacearum* infection to potato (*Solanum commersonii*) was also found to induce genes related to SA, ET, biotic stress and cell wall modification (Zuluaga et al., 2015). Narancio et al. (2013) also reported the *R. solanacearum* defense in potato (*S. commersonii* Dun) to be mediated by ET and SA mediated responses. They also reported up-regulation of *ERF, PR*, and *WRKY* genes. It was evident through transcriptome of LD-pathogen that *R. solanacearum* alone induces drought like symptoms. LD-pathogen stress showed highly up-regulated expression of genes encoding RAFFINOSE SYNTHASE, LEA14, MYOINOSITOL OXYGENASE, and CPRF2. The transcriptomic responses were close to SD-combined stress transcriptome indicating that chickpea upon *R. solanacearum* infection feels drought stress like effect. Xylem invading *Xylella fastidiosa* was also found to invoke drought like response as it up-regulated expression ABA biosynthesis genes and two galactinol synthase genes involved in synthesis of galactinol and raffinose osmoprotectants (Choi et al., 2013).

The transcriptomic changes under SD-combined stress in this study directed toward defense response mediated SA and ET signaling and lignin and flavonoid accumulation. Contrastingly, LD-combined stress showed repressed expression of SA and ET signaling genes along with various defense related genes and thus induced the susceptibility of plant. We noted differential expression of various genes unique to SD- and LD-combined stresses. One of the unique responses under SD-combined stress was specific up-regulation of ET signaling gene Multiprotein Bridging Factor-1c (*MBF1c*). MBF1 is a DNA-binding protein transcriptional coactivator which is involved in regulating metabolic and development pathways (Suzuki et al., 2011). Earlier, up-regulation of *MBF1c* was noticed in *A. thaliana* under pathogen infection, salinity, drought, heat, hydrogen peroxide, ABA, and SA application (Rizhsky et al., 2004; Tsuda and Yamazaki, 2004). Moreover, its constitutive expression was reported to increase the tolerance of transgenic plants to bacterial infection, salinity, heat, and osmotic stress and combined heat and osmotic stress (Suzuki et al., 2005). Similarly, genes encoding for auxin receptor TIR, transporters ABCB4, HP4, ARR17, PXY, and SND2 showed up-regulation only under SD-combined stress.

Xylem being conductor of water in plant is the most affected tissue under drought (De Souza et al., 2013) and wilt diseases (Yadeta and Thomma, 2013). While inhabiting the xylem, vascular pathogen exploits all inorganic and sugar resources present in the xylem (Yadeta and Thomma, 2013). Also, it enzymatically digests xylem cell wall to fulfill its nutritional needs (Yadeta and Thomma, 2013). The plant in turn, induces vascular coating as well as metabolic changes like secretion of PR proteins, peroxidases, proteases, xyloglucan-endotransglycosylase (XET), and xyloglucan-specific endoglucanase inhibitor protein (XEGIP), phenols, phytoalexins, and lignin-like compounds as a part of the plant defense toward the pathogen (Yadeta and Thomma, 2013). Primary and SCW also modulate ET, JA, SA, and ABA hormone signaling and thus have role in drought stress tolerance and regulation of defense response (Schulze-Lefert, 2004; Somerville et al., 2004; Hernández-Blanco et al., 2007; Denancé et al., 2013). In this regard, we looked for the expression of genes involved in xylem differentiation and SCW modification to understand if plant is utilizing xylem modification or re-differentiation as defense mechanism against combined stress. We observed that SD-combined stress had up-regulated expression of genes involved in xylem differentiation and SCW modification especially genes involved in lignin biosynthesis. During LD-combined stress the genes involved in cellulose biosynthesis were up-regulated. Increased lignin accumulation has been shown to increase the plants defense mechanism (Bhuiyan et al., 2009; Xu et al., 2011). However, on contrary increased cellulose synthesis is known to increase the susceptibility of plants toward pathogen. Hernández-Blanco et al. (2007) showed that mutation in cellulose synthase encoding genes (*CeSA4, CeSA7*, and *CeSA8*) for cellulose deposition in SCW enhanced resistance of *A. thaliana* toward *R. solanacearum*. In our study, we could partly correlate increased lignification during SD-combined stress with the enhanced defense under this stress and up-regulation of *CeSA7* with the compromised disease resistance in LD-combined stressed plants.

## Conclusion

The study highlights that combined drought and *R. solanacearum* stress invokes transcriptome changes unique to combined stress and also transcriptome common between combined and individual stresses in chickpea. SD-combined stress in chickpea causes up-regulation of genes involved in SA, ET signaling, and lignin biosynthesis. LD-combined stress down-regulates the expression of defense related genes and increases expression of genes involved in cellulose biosynthesis resulting in susceptibility of chickpea toward *R. solanacearum*. Transcriptome under *R. solanacearum* infection exhibit up-regulated expression of various abiotic stress related genes and displays closeness with the SD-combined stress transcriptome.

## Author Contributions

MS-K conceived the idea. MS-K and RS designed the study. RS and AG performed the experiments. RS analyzed the data with the input from MS-K. MS-K and RS wrote the manuscript.

## Conflict of Interest Statement

The authors declare that the research was conducted in the absence of any commercial or financial relationships that could be construed as a potential conflict of interest.

## Footnotes

1 http://www.nipgr.res.in/ctdb.html

2 https://software.broadinstitute.org/morpheus/

3 http://bioinfogp.cnb.csic.es/tools/venny/

4 http://www.nipgr.res.in/ctdb.html

5 http://bioinfo.cau.edu.cn/agriGO/analysis.php

6 http://www.broadinstitute.org/cancer/software/GENE-E/

7 http://www.nipgr.res.in/ctdb.html

8 https://blast.ncbi.nlm.nih.gov/Blast.cgi

9 http://bbc.botany.utoronto.ca/efp/cgi-bin/efpWeb.cgi
