# Supplementary material for: Concurrent Drought Stress and Vascular Pathogen Infection Induce Common and Distinct Transcriptomic Responses in Chickpea
Source: Front Plant Sci. 2017 Mar 14;8:333. doi: 10.3389/fpls.2017.00333 (PMC5361651; doi:10.3389/fpls.2017.00333)
Supplement: Supplementary file 1 [file Table_1.DOCX]

| S. No. | Gene IDs | Gene name | Primer Sequence (5’ to 3’) |
| --- | --- | --- | --- |
| 1 | TC32277 | Eukaryotic aspartyl protease protein 1 | FP: TCTGAAGCCAAAGCAGAAGCA |
|  |  |  | RP: TTGGGTACCCTGTGATTGCAC |
| 2 | TC22121 | Phosphatidylinositol polyphosphate 5-phosphatase 11 | FP: CTGGCTTATGATCCGAACCGT |
|  |  |  | RP: GGTTCGAGTACCAGCATGGA |
| 3 | TC06168 | 3-oxo-Delta(4,5)-steroid 5-beta-reductase | FP: AATGCTCCGAATCTTCGCCA |
|  |  |  | RP: TTGAGTATGTCGAGGCGTGG |
| 4 | TC03424 | Myosin heavy chain kinase C | FP: TCGTATTTAGCGGTTCAGCC |
|  |  |  | RP: GTTCTTCAACGGCAATGCAC |
| 5 | TC11095 | Isopiperitenol-carveol dehydrogenase | FP: ACGTGTGGTCGTCATAGCTG |
|  |  |  | RP: GATCTTCGTCTGCCACGTCA |
| 6 | TC01043 | Uncharacterized | FP:CACCGGTGCTATTGCTGTTG |
|  |  |  | RP:TGAGATGGATCTACTGCGGC |
| 7 | TC07115 | Vicilin-like | FP:AAGAGAACGAGGTCGCCAAG |
|  |  |  | RP:CATGGTGAGGATGACGCTGT |
| 8 | TC10598 | Proline dehydrogenase 2 | FP:CAACTCGCGTGGTCCC |
|  |  |  | RP:AGGTCGACCGTTGAAGTGAC |
| 9 | TC17224 | Beta-glucosidase 12-like | FP:ATCGTCCAGGTGCTAACTGC |
|  |  |  | RP:TTCGAGACTATGCGGAGCTT |
| 10 | TC22250 | Laccase-7-like | FP:TGGTGCCTCCCAAACAACAC |
|  |  |  | RP:CGTGGAACTGGTACCCAATGT |
| 11 | TC28275 | Uncharacterized acetyltransferase | FP:ACCATGGTTGCGTTTGGGA |
|  |  |  | RP:TGAAACTATGGCGCTGCTTG |
| 12 | TC33834 | Reticuline oxidase-like protein | FP:ACCAAGAAATGAATAAGTCACCAAC |
|  |  |  | RP:TGAACAAAGTATCCCTATTCTGCC |
| 13 | TC34213 | Dirigent protein 22-like | FP:CAATGGTAGCATCCCCCGAT |
|  |  |  | RP:GCCTGTGATTGGTGGAAGTG |
| 14 | TC13198 | Cytochrome c oxidase subunit 2 | FP:TCTTGCTCGAAGGGTAAGCG |
|  |  |  | RP:CCCGCTAACCGACAACTGAG |
| 15 | EU529707.1 | CaActin | FP: GCCCGCGACGTTGTGA |
|  |  |  | RP:CCTTGTTACGACTTCTCCTTCCTCTA |

**Supplementary Table S1:** List of primers used for real-time PCR.

**FP: Forward primer; RP: Reverse primer**

**Gene IDs TCXXXXX is chickpea gene identifier from chickpea transcriptome database (CTDB)**

**Gene ID EU529707.1 is GenBank accession**
