# Supplementary material for: Concurrent Drought Stress and Vascular Pathogen Infection Induce Common and Distinct Transcriptomic Responses in Chickpea
Source: Front Plant Sci. 2017 Mar 14;8:333. doi: 10.3389/fpls.2017.00333 (PMC5361651; doi:10.3389/fpls.2017.00333)
Supplement: Supplementary file 2 [file Table_2.DOCX]

**Supplementary Table S2**: Number and percentage of unannotated genes in total differentially expressed genes

| Treatments | Number of unannotated genes | Total number of DEGs | % of unannotated DEGs |
| --- | --- | --- | --- |
| SD-combined up-regulated | 170 | 487 | 34.9076 |
| SD-combined down-regulated | 128 | 523 | 24.47419 |
| SD-drought up-regulated | 150 | 403 | 37.22084 |
| SD-drought down-regulated | 250 | 775 | 32.25806 |
| SD-pathogen up-regulated | 122 | 298 | 40.9396 |
| SD-pathogen down-regulated | 67 | 307 | 21.8241 |
| LD-combined up-regulated | 206 | 553 | 37.25136 |
| LD-combined down-regulated | 182 | 734 | 24.79564 |
| LD-drought up-regulated | 201 | 565 | 35.57522 |
| LD-drought down-regulated | 255 | 851 | 29.96475 |
| LD-pathogen up-regulated | 166 | 458 | 36.24454 |
| LD-pathogen down-regulated | 121 | 435 | 27.81609 |
