# Supplementary material for: Concurrent Drought Stress and Vascular Pathogen Infection Induce Common and Distinct Transcriptomic Responses in Chickpea
Source: Front Plant Sci. 2017 Mar 14;8:333. doi: 10.3389/fpls.2017.00333 (PMC5361651; doi:10.3389/fpls.2017.00333)
Supplement: Supplementary file 3 [file Table_3.DOCX]

| Supplementary Table S3: Number of DEGs with Arabidopsis thaliana gene IDs out of total DEGs under each stress category | | | | | | | |
| --- | --- | --- | --- | --- | --- | --- | --- |
|  | **Up-regulated** | | |  | **Down-regulated** | | |
| Treatments | **No of genes with TAIR ID** | **Total DEGs** | **% DEGs with TAIR ID** |  | **No of genes with TAIR ID** | **Total DEGs** | **% DEGs with TAIR ID** |
| SD-pathogen | 170 | 298 | 57.04698 |  | 238 | 307 | 77.52443 |
| SD-drought | 235 | 403 | 58.31266 |  | 484 | 775 | 62.45161 |
| SD-combined | 289 | 487 | 59.34292 |  | 363 | 523 | 69.40727 |
| LD-pathogen | 260 | 458 | 56.76856 |  | 260 | 435 | 59.77011 |
| LD-drought | 310 | 565 | 54.86726 |  | 550 | 851 | 64.62985 |
| LD-combined | 323 | 553 | 58.40868 |  | 498 | 734 | 67.84741 |
