# Supplementary material for: Concurrent Drought Stress and Vascular Pathogen Infection Induce Common and Distinct Transcriptomic Responses in Chickpea
Source: Front Plant Sci. 2017 Mar 14;8:333. doi: 10.3389/fpls.2017.00333 (PMC5361651; doi:10.3389/fpls.2017.00333)
Supplement: Supplementary file 4 [file Presentation_1.pptx]

## Slide 1
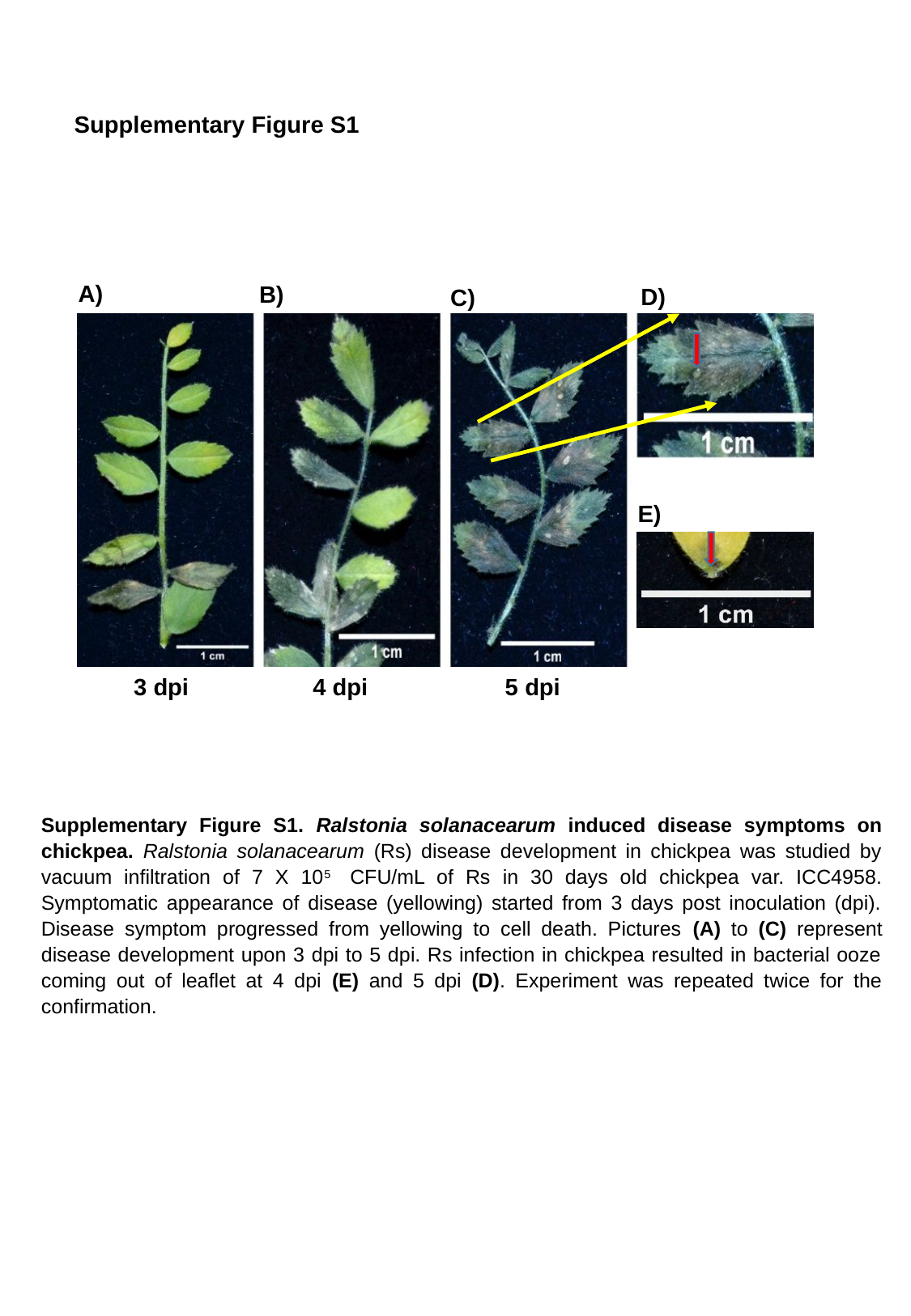

Supplementary Figure S1
A)
B)
D)
C)
E)
3 dpi
4 dpi
5 dpi
Supplementary Figure S1. Ralstonia solanacearum induced disease symptoms on chickpea. Ralstonia solanacearum (Rs) disease development in chickpea was studied by vacuum infiltration of 7 X 105 CFU/mL of Rs in 30 days old chickpea var. ICC4958. Symptomatic appearance of disease (yellowing) started from 3 days post inoculation (dpi). Disease symptom progressed from yellowing to cell death. Pictures (A) to (C) represent disease development upon 3 dpi to 5 dpi. Rs infection in chickpea resulted in bacterial ooze coming out of leaflet at 4 dpi (E) and 5 dpi (D). Experiment was repeated twice for the confirmation.

## Slide 2
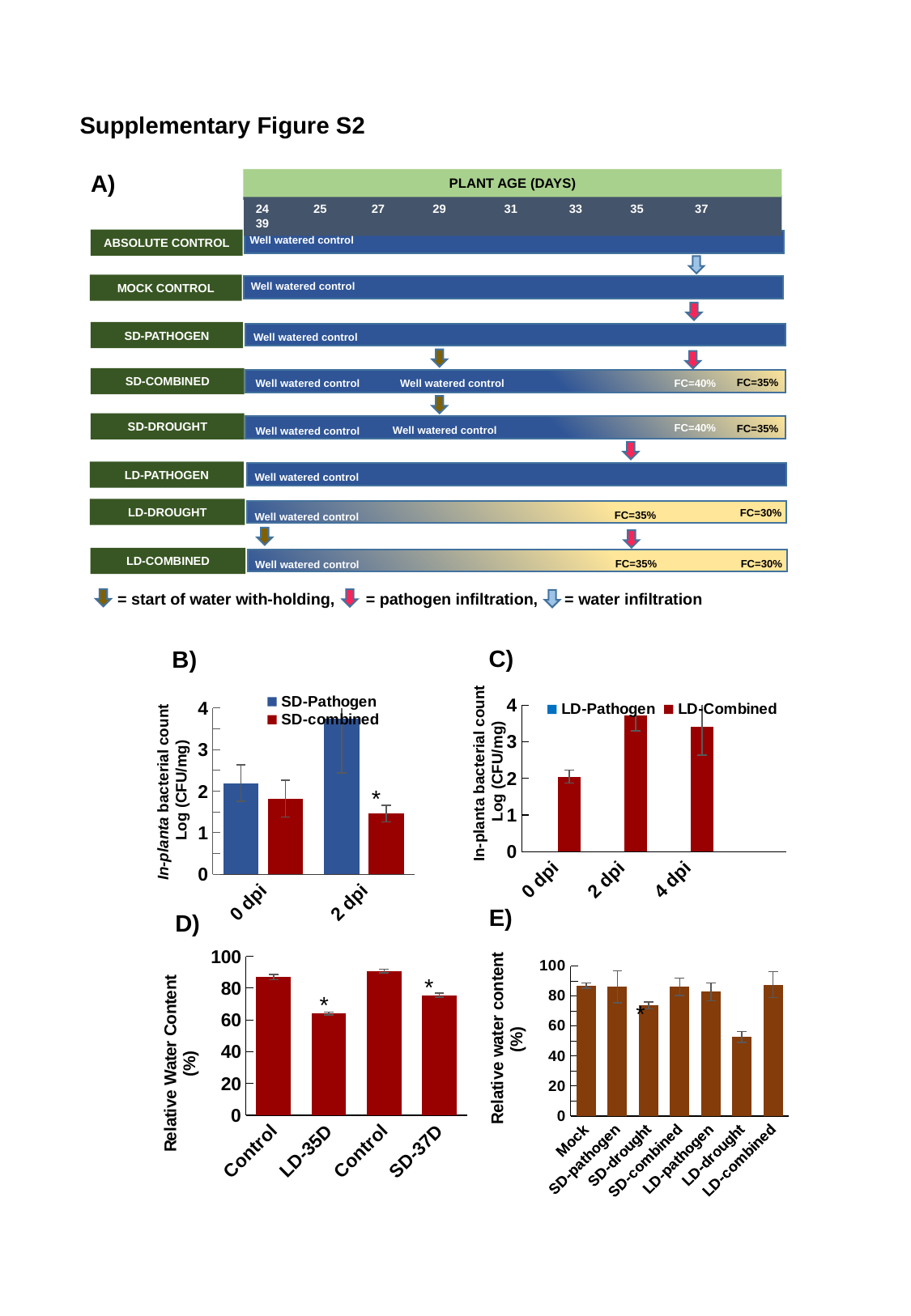

Supplementary Figure S2
A)
PLANT AGE (DAYS)
24 25 27 29 31 33 35 37 39
Well watered control
ABSOLUTE CONTROL
Well watered control
MOCK CONTROL
SD-PATHOGEN
Well watered control
SD-COMBINED
FC=35%
FC=40%
Well watered control
Well watered control
SD-DROUGHT
FC=40%
FC=35%
Well watered control
Well watered control
LD-PATHOGEN
Well watered control
LD-DROUGHT
FC=30%
FC=35%
Well watered control
LD-COMBINED
FC=35%
FC=30%
Well watered control
 = start of water with-holding, = pathogen infiltration, = water infiltration
C)
B)
### Chart
| Category | LD-Pathogen | LD-Combined |
|---|---|---|
| 0 dpi | 1.9952442009592306 | 2.0471097175988224 |
| 2 dpi | 4.545763361796822 | 3.716968830971778 |
| 4 dpi | 3.474359416841306 | 3.4019627767024905 |In-planta bacterial count
Log (CFU/mg)
### Chart
| Category | SD-Pathogen | SD-combined |
|---|---|---|
| 0 dpi | 2.1902297404596136 | 1.8181940100539276 |
| 2 dpi | 3.7483907260663285 | 1.4589169032006997 |In-planta bacterial count
Log (CFU/mg)
*
E)
D)
### Chart
| Category | |
|---|---|
| Control | 86.97139063440842 |
| LD-35D | 64.08603478635402 |
| Control | 90.70165638509741 |
| SD-37D | 75.5491597194654 |*
*
### Chart
| Category | |
|---|---|
| Mock | 86.97139063440841 |
| SD-pathogen | 86.08115942028986 |
| SD-drought | 73.7692201950734 |
| SD-combined | 86.10075870552488 |
| LD-pathogen | 82.80294759825324 |
| LD-drought | 52.60795454545455 |
| LD-combined | 87.51088534107402 |*

## Slide 3
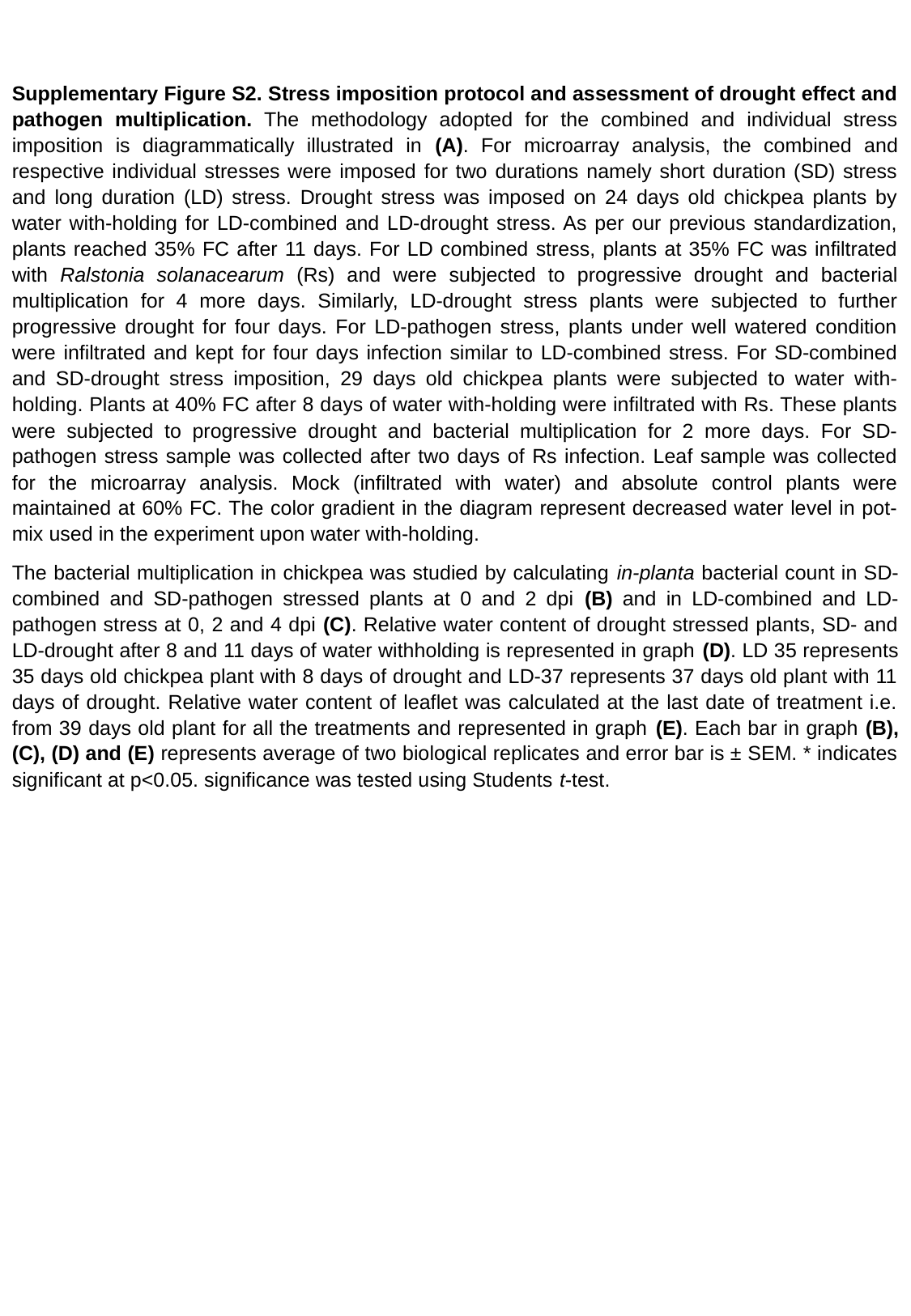

Supplementary Figure S2. Stress imposition protocol and assessment of drought effect and pathogen multiplication. The methodology adopted for the combined and individual stress imposition is diagrammatically illustrated in (A). For microarray analysis, the combined and respective individual stresses were imposed for two durations namely short duration (SD) stress and long duration (LD) stress. Drought stress was imposed on 24 days old chickpea plants by water with-holding for LD-combined and LD-drought stress. As per our previous standardization, plants reached 35% FC after 11 days. For LD combined stress, plants at 35% FC was infiltrated with Ralstonia solanacearum (Rs) and were subjected to progressive drought and bacterial multiplication for 4 more days. Similarly, LD-drought stress plants were subjected to further progressive drought for four days. For LD-pathogen stress, plants under well watered condition were infiltrated and kept for four days infection similar to LD-combined stress. For SD-combined and SD-drought stress imposition, 29 days old chickpea plants were subjected to water with-holding. Plants at 40% FC after 8 days of water with-holding were infiltrated with Rs. These plants were subjected to progressive drought and bacterial multiplication for 2 more days. For SD-pathogen stress sample was collected after two days of Rs infection. Leaf sample was collected for the microarray analysis. Mock (infiltrated with water) and absolute control plants were maintained at 60% FC. The color gradient in the diagram represent decreased water level in pot-mix used in the experiment upon water with-holding.
The bacterial multiplication in chickpea was studied by calculating in-planta bacterial count in SD-combined and SD-pathogen stressed plants at 0 and 2 dpi (B) and in LD-combined and LD-pathogen stress at 0, 2 and 4 dpi (C). Relative water content of drought stressed plants, SD- and LD-drought after 8 and 11 days of water withholding is represented in graph (D). LD 35 represents 35 days old chickpea plant with 8 days of drought and LD-37 represents 37 days old plant with 11 days of drought. Relative water content of leaflet was calculated at the last date of treatment i.e. from 39 days old plant for all the treatments and represented in graph (E). Each bar in graph (B), (C), (D) and (E) represents average of two biological replicates and error bar is ± SEM. * indicates significant at p<0.05. significance was tested using Students t-test.

## Slide 4
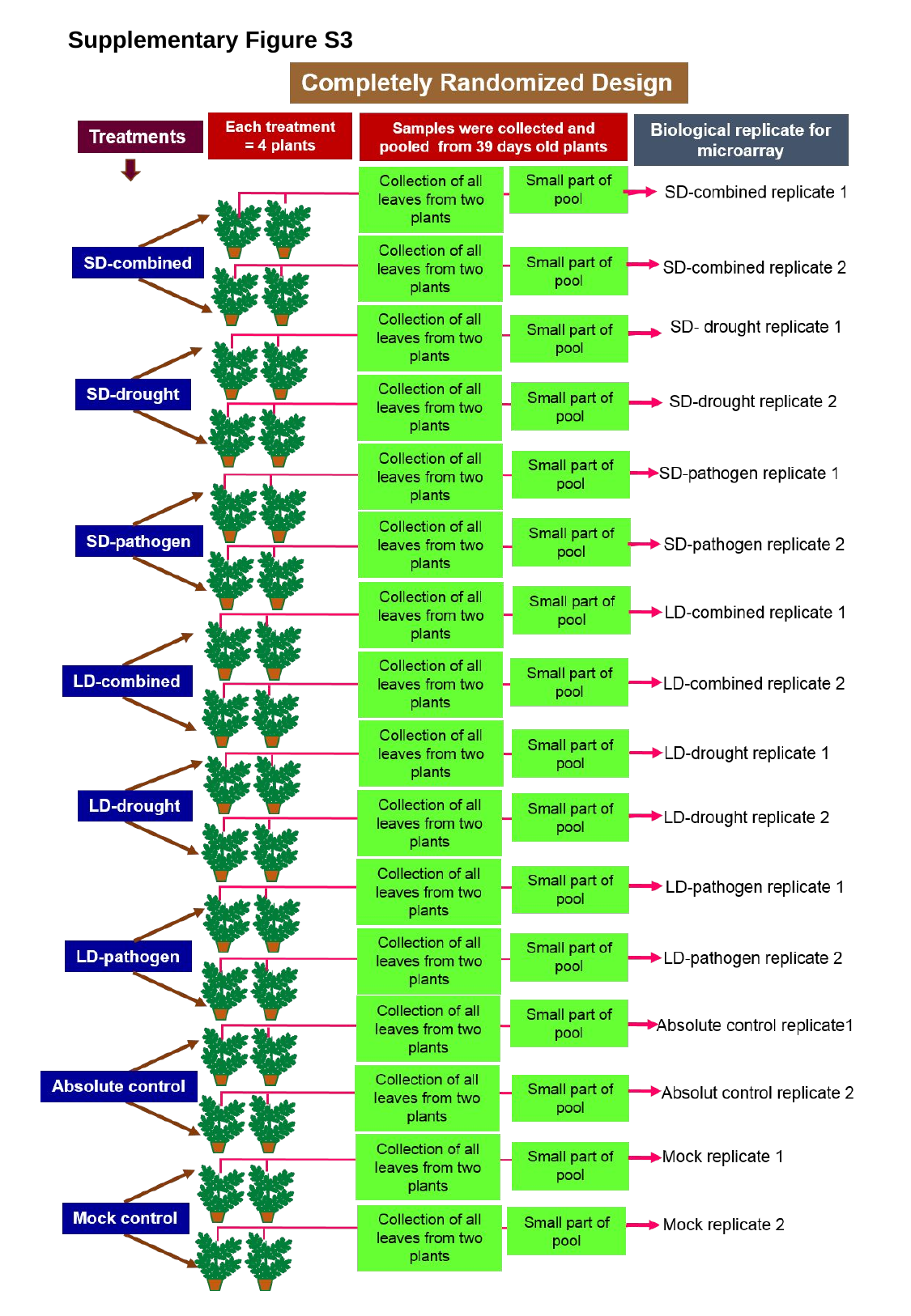

Supplementary Figure S3

## Slide 5
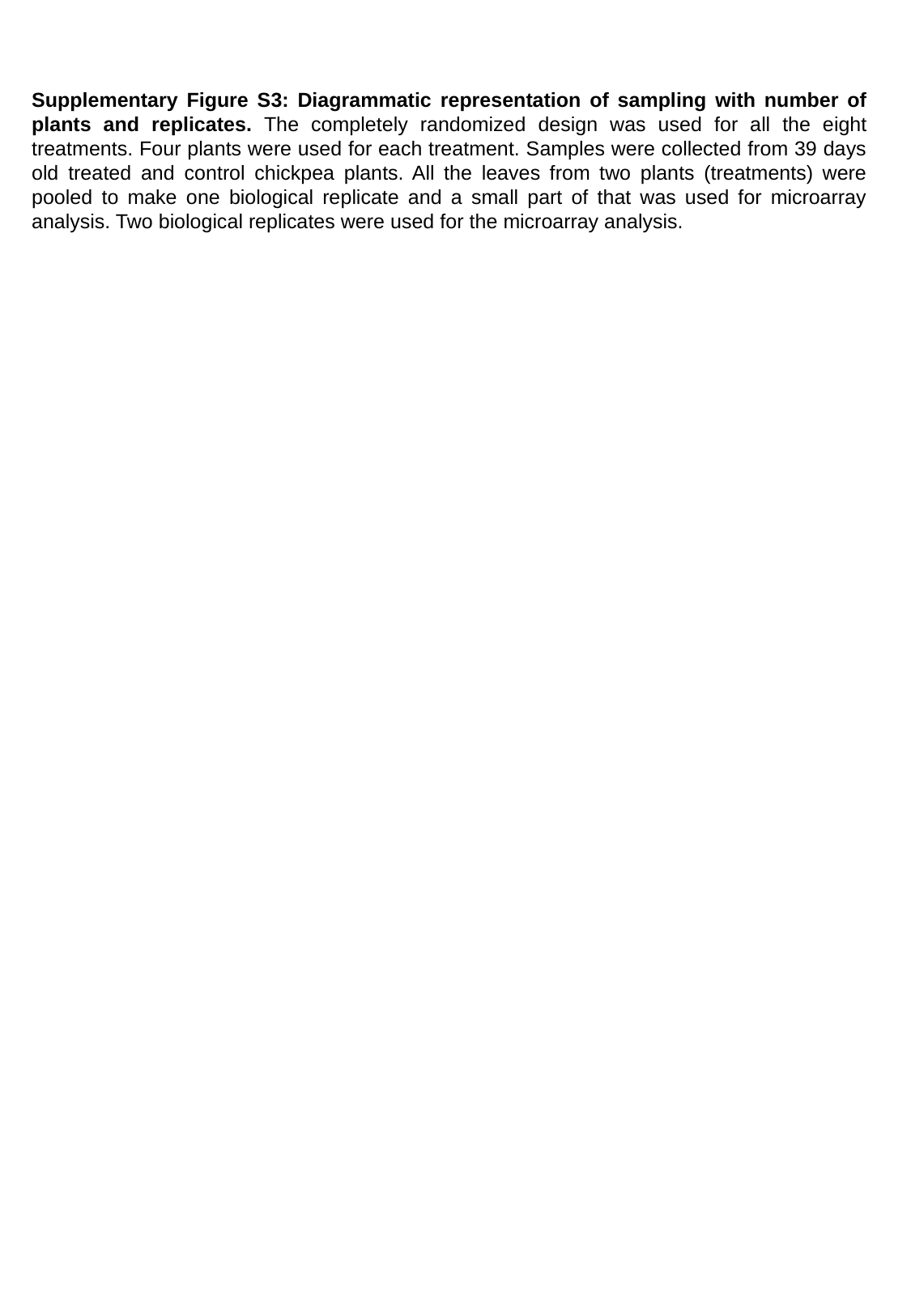

Supplementary Figure S3: Diagrammatic representation of sampling with number of plants and replicates. The completely randomized design was used for all the eight treatments. Four plants were used for each treatment. Samples were collected from 39 days old treated and control chickpea plants. All the leaves from two plants (treatments) were pooled to make one biological replicate and a small part of that was used for microarray analysis. Two biological replicates were used for the microarray analysis.

## Slide 6
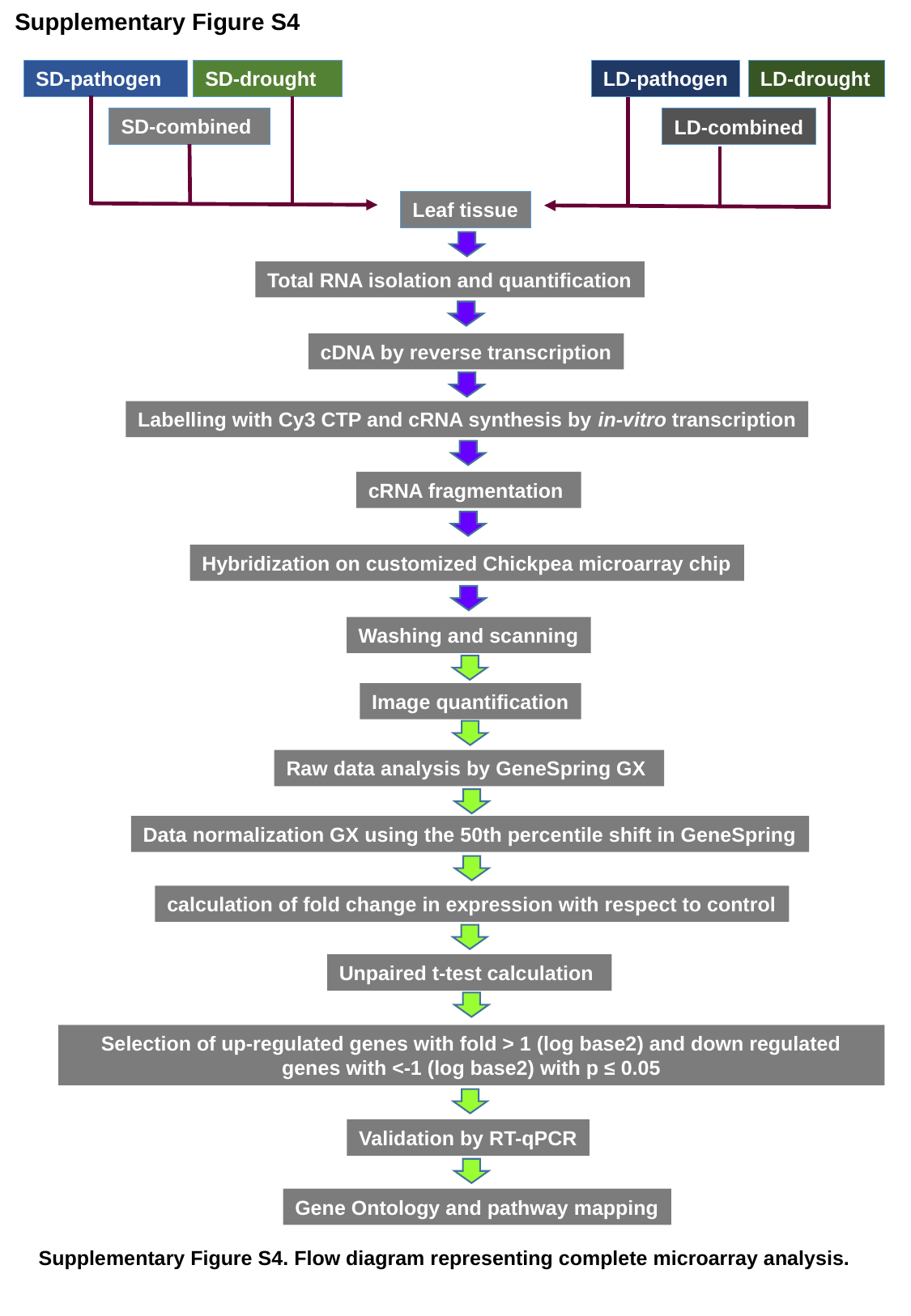

Supplementary Figure S4
SD-pathogen
LD-pathogen
LD-drought
SD-drought
SD-combined
LD-combined
Leaf tissue
Total RNA isolation and quantification
cDNA by reverse transcription
Labelling with Cy3 CTP and cRNA synthesis by in-vitro transcription
cRNA fragmentation
Hybridization on customized Chickpea microarray chip
Washing and scanning
Image quantification
Raw data analysis by GeneSpring GX
Data normalization GX using the 50th percentile shift in GeneSpring
calculation of fold change in expression with respect to control
Unpaired t-test calculation
Selection of up-regulated genes with fold > 1 (log base2) and down regulated genes with <-1 (log base2) with p ≤ 0.05
Validation by RT-qPCR
Gene Ontology and pathway mapping
Supplementary Figure S4. Flow diagram representing complete microarray analysis.

## Slide 7
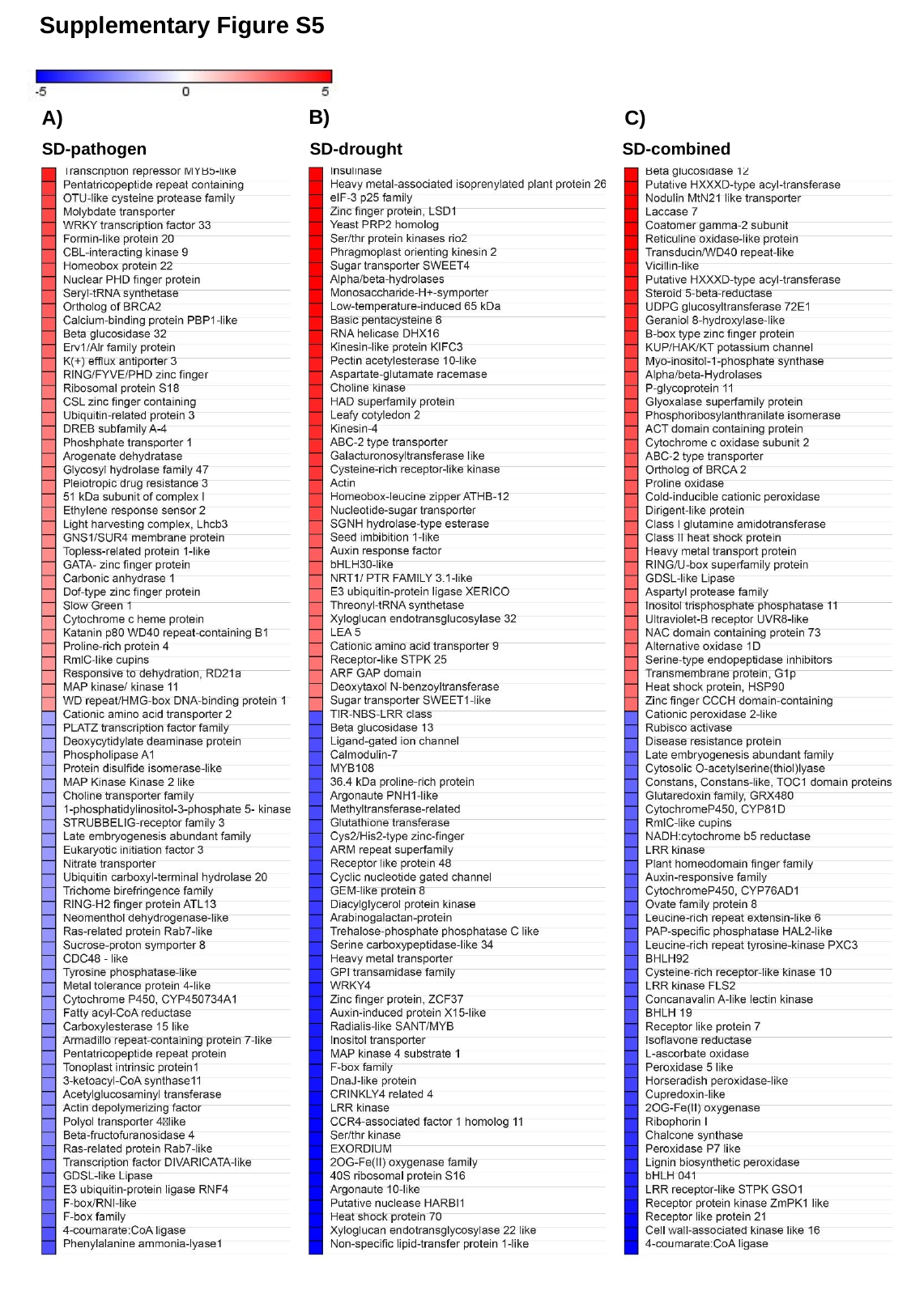

Supplementary Figure S5
B)
C)
A)
SD-combined
SD-pathogen
SD-drought

## Slide 8
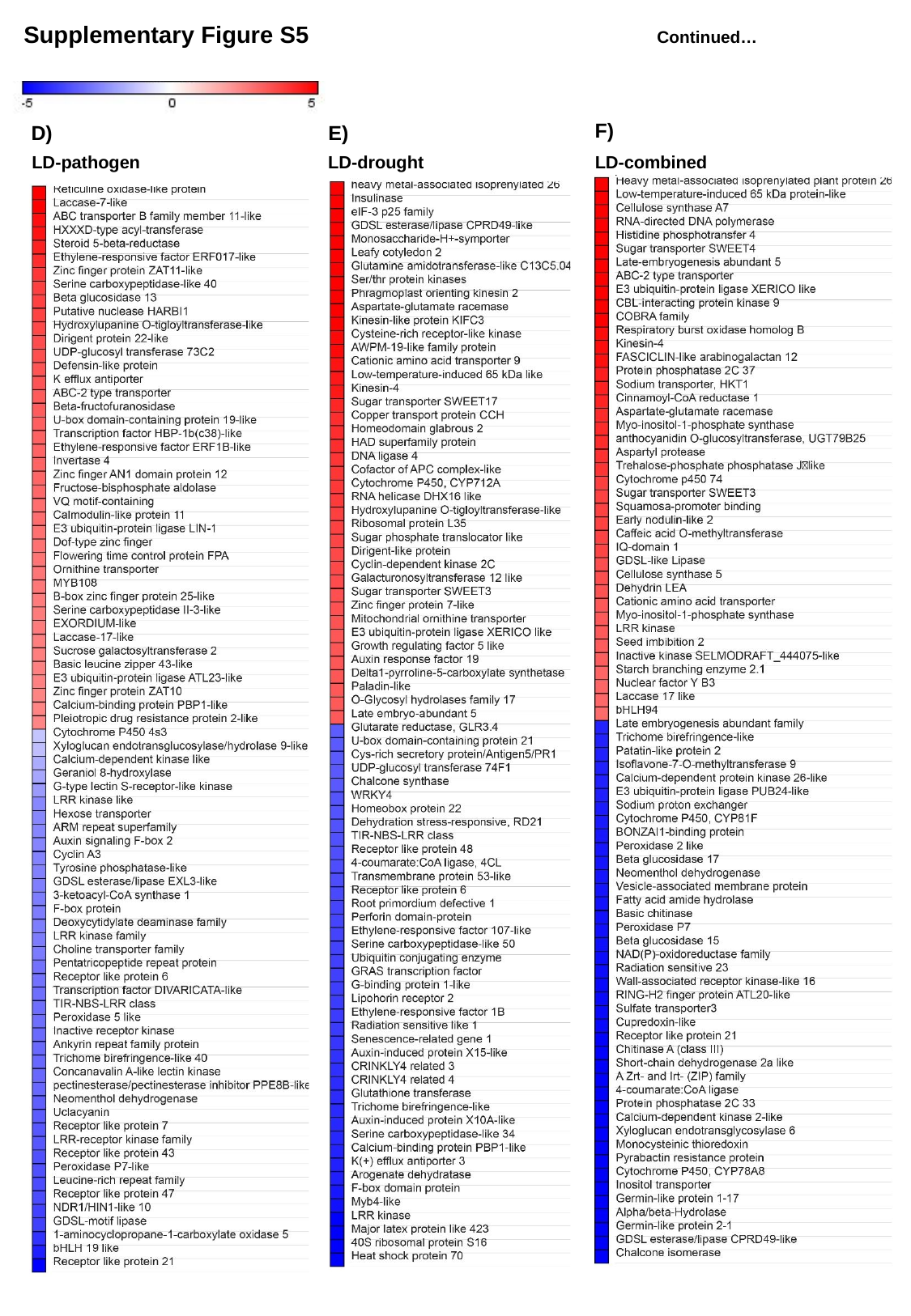

Supplementary Figure S5
Continued…
F)
E)
D)
LD-pathogen
LD-drought
LD-combined

## Slide 9
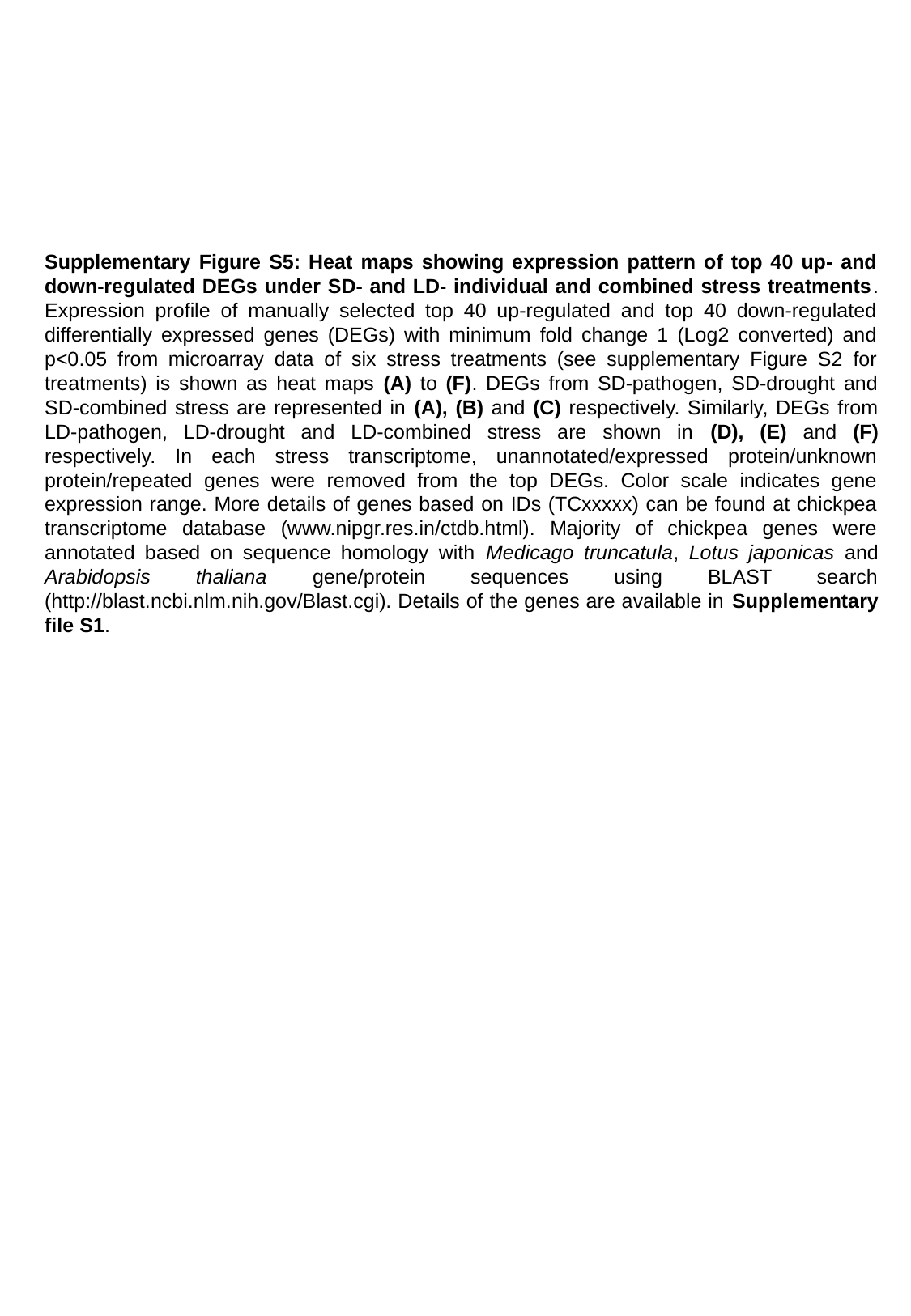

Supplementary Figure S5: Heat maps showing expression pattern of top 40 up- and down-regulated DEGs under SD- and LD- individual and combined stress treatments. Expression profile of manually selected top 40 up-regulated and top 40 down-regulated differentially expressed genes (DEGs) with minimum fold change 1 (Log2 converted) and p<0.05 from microarray data of six stress treatments (see supplementary Figure S2 for treatments) is shown as heat maps (A) to (F). DEGs from SD-pathogen, SD-drought and SD-combined stress are represented in (A), (B) and (C) respectively. Similarly, DEGs from LD-pathogen, LD-drought and LD-combined stress are shown in (D), (E) and (F) respectively. In each stress transcriptome, unannotated/expressed protein/unknown protein/repeated genes were removed from the top DEGs. Color scale indicates gene expression range. More details of genes based on IDs (TCxxxxx) can be found at chickpea transcriptome database (www.nipgr.res.in/ctdb.html). Majority of chickpea genes were annotated based on sequence homology with Medicago truncatula, Lotus japonicas and Arabidopsis thaliana gene/protein sequences using BLAST search (http://blast.ncbi.nlm.nih.gov/Blast.cgi). Details of the genes are available in Supplementary file S1.

## Slide 10
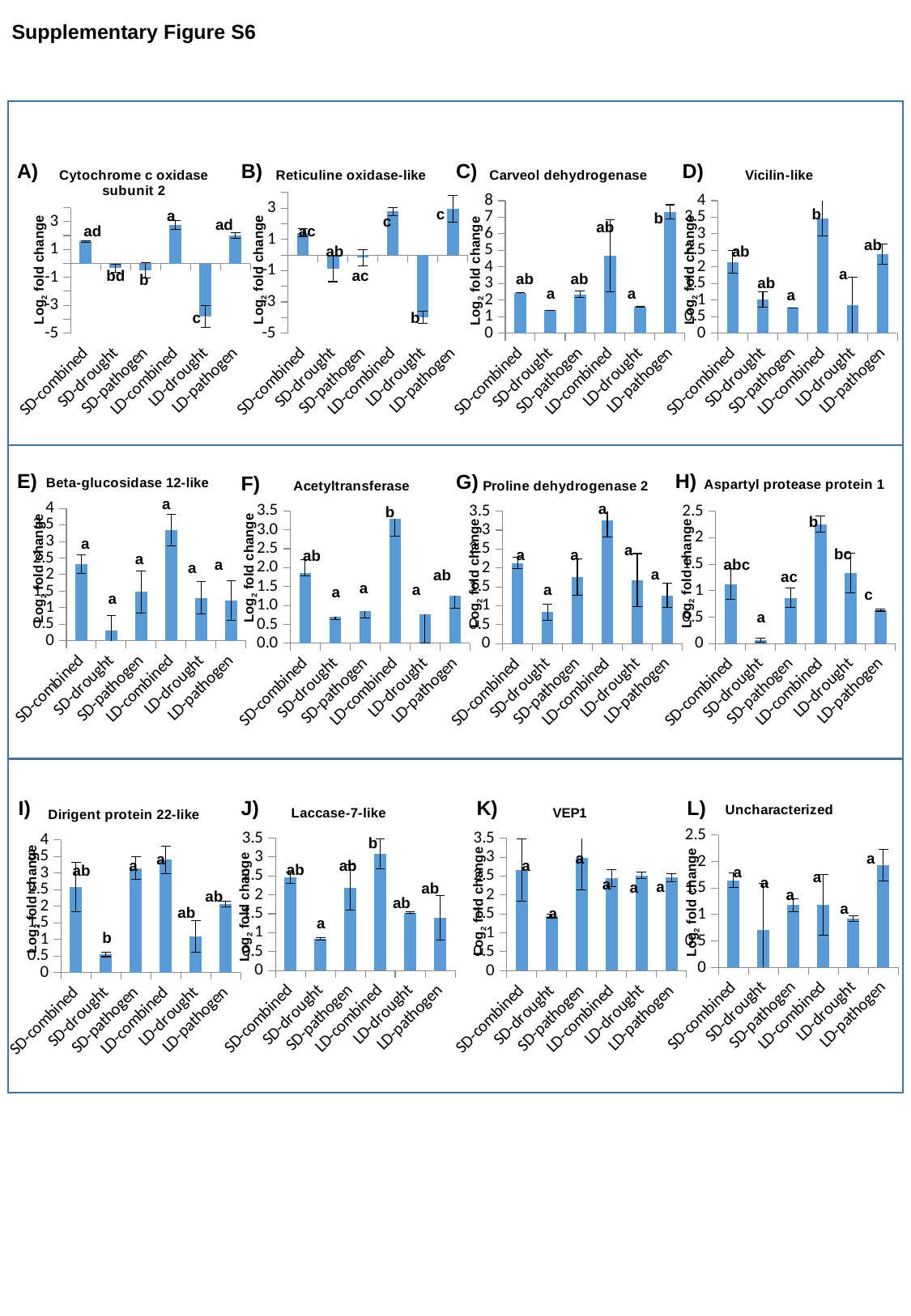

Supplementary Figure S6
### Chart: Cytochrome c oxidase subunit 2
| Category | TC13198 Cytochrome c oxidase subunit 2 |
|---|---|
| SD-combined | 1.565250396728516 |
| SD-drought | -0.3290004730224614 |
| SD-pathogen | -0.4820003509521486 |
| LD-combined | 2.7635002136230478 |
| LD-drought | -3.789501667022706 |
| LD-pathogen | 2.009249687194823 |Log2 fold change
### Chart: Reticuline oxidase-like
| Category | TC33834 Reticuline oxidase-like |
|---|---|
| SD-combined | 1.4232501983642578 |
| SD-drought | -0.8720006942749026 |
| SD-pathogen | -0.1752505302429201 |
| LD-combined | 2.77274990081787 |
| LD-drought | -3.996751308441162 |
| LD-pathogen | 2.9475002288818373 |Log2 fold change
### Chart: Carveol dehydrogenase
| Category | TC11095 Carveol dehydrogenase |
|---|---|
| SD-combined | 2.4427504539489737 |
| SD-drought | 1.378249168395996 |
| SD-pathogen | 2.3472490310668936 |
| LD-combined | 4.693999290466309 |
| LD-drought | 1.5902481079101562 |
| LD-pathogen | 7.334749221801758 |Log2 fold change
### Chart: Vicilin-like
| Category | TC07115 Vicilin-like  |
|---|---|
| SD-combined | 2.1550002098083487 |
| SD-drought | 1.016000270843506 |
| SD-pathogen | 0.7617492675781258 |
| LD-combined | 3.4685001373291007 |
| LD-drought | 0.8352484703063968 |
| LD-pathogen | 2.3902502059936523 |Log2 fold change
A)
B)
C)
D)
b
c
a
b
c
ad
ab
ad
ac
ab
ab
ab
a
bd
ac
ab
ab
b
ab
a
a
a
c
b
### Chart: Beta-glucosidase 12-like
| Category | TC17224 Beta-glucosidase 12-like  |
|---|---|
| SD-combined | 2.3067498207092267 |
| SD-drought | 0.31724977493286144 |
| SD-pathogen | 1.4752492904663081 |
| LD-combined | 3.342750072479247 |
| LD-drought | 1.2942490577697754 |
| LD-pathogen | 1.2182497978210445 |Log2 fold change
### Chart: Acetyltransferase
| Category | TC28275 Acetyltransferase |
|---|---|
| SD-combined | 1.866250038146973 |
| SD-drought | 0.7099995613098148 |
| SD-pathogen | 0.8552489280700686 |
| LD-combined | 3.287750244140626 |
| LD-drought | 0.7609982490539552 |
| LD-pathogen | 1.2627501487731938 |Log2 fold change
H)
### Chart: Proline dehydrogenase 2
| Category | TC10598 Proline dehydrogenase 2 |
|---|---|
| SD-combined | 2.1292500495910645 |
| SD-drought | 0.8317499160766606 |
| SD-pathogen | 1.7547497749328613 |
| LD-combined | 3.2577505111694345 |
| LD-drought | 1.6764993667602541 |
| LD-pathogen | 1.2702498435974119 |Log2 fold change
### Chart: Aspartyl protease protein 1
| Category | TC32277 Aspartyl protease protein 1 |
|---|---|
| SD-combined | 1.1255002021789546 |
| SD-drought | 0.060750007629394656 |
| SD-pathogen | 0.863749980926514 |
| LD-combined | 2.2552499771118164 |
| LD-drought | 1.3330011367797856 |
| LD-pathogen | 0.635499477386475 |Log2 fold change
E)
G)
F)
a
a
b
b
a
a
bc
a
a
ab
a
a
abc
a
a
ab
ac
a
a
a
a
c
a
a
### Chart: Uncharacterized
| Category | TC01043 Uncharacterized  |
|---|---|
| SD-combined | 1.645500659942627 |
| SD-drought | 0.7109990119934086 |
| SD-pathogen | 1.174749851226807 |
| LD-combined | 1.1774992942810059 |
| LD-drought | 0.9214992523193362 |
| LD-pathogen | 1.9262490272521973 |Log2 fold change
I)
J)
K)
L)
### Chart: Laccase-7-like
| Category | TC22250 Laccase-7-like |
|---|---|
| SD-combined | 2.456000804901122 |
| SD-drought | 0.8342499732971194 |
| SD-pathogen | 2.192500114440918 |
| LD-combined | 3.087751388549805 |
| LD-drought | 1.529749393463134 |
| LD-pathogen | 1.4007506370544427 |Log2 fold change
### Chart: VEP1
| Category | VEP1 |
|---|---|
| SD-combined | 2.658250331878661 |
| SD-drought | 1.4509997367858882 |
| SD-pathogen | 2.976000785827637 |
| LD-combined | 2.4472503662109384 |
| LD-drought | 2.514501094818115 |
| LD-pathogen | 2.4659996032714844 |Log2 fold change
### Chart: Dirigent protein 22-like
| Category | TC34213 Dirigent protein 22-like |
|---|---|
| SD-combined | 2.5737500190734863 |
| SD-drought | 0.5442490577697757 |
| SD-pathogen | 3.1517486572265625 |
| LD-combined | 3.400499343872071 |
| LD-drought | 1.0927481651306157 |
| LD-pathogen | 2.071249485015867 |Log2 fold change
b
a
a
a
ab
a
a
ab
ab
a
a
a
a
a
a
ab
a
ab
ab
a
ab
a
a
b

## Slide 11
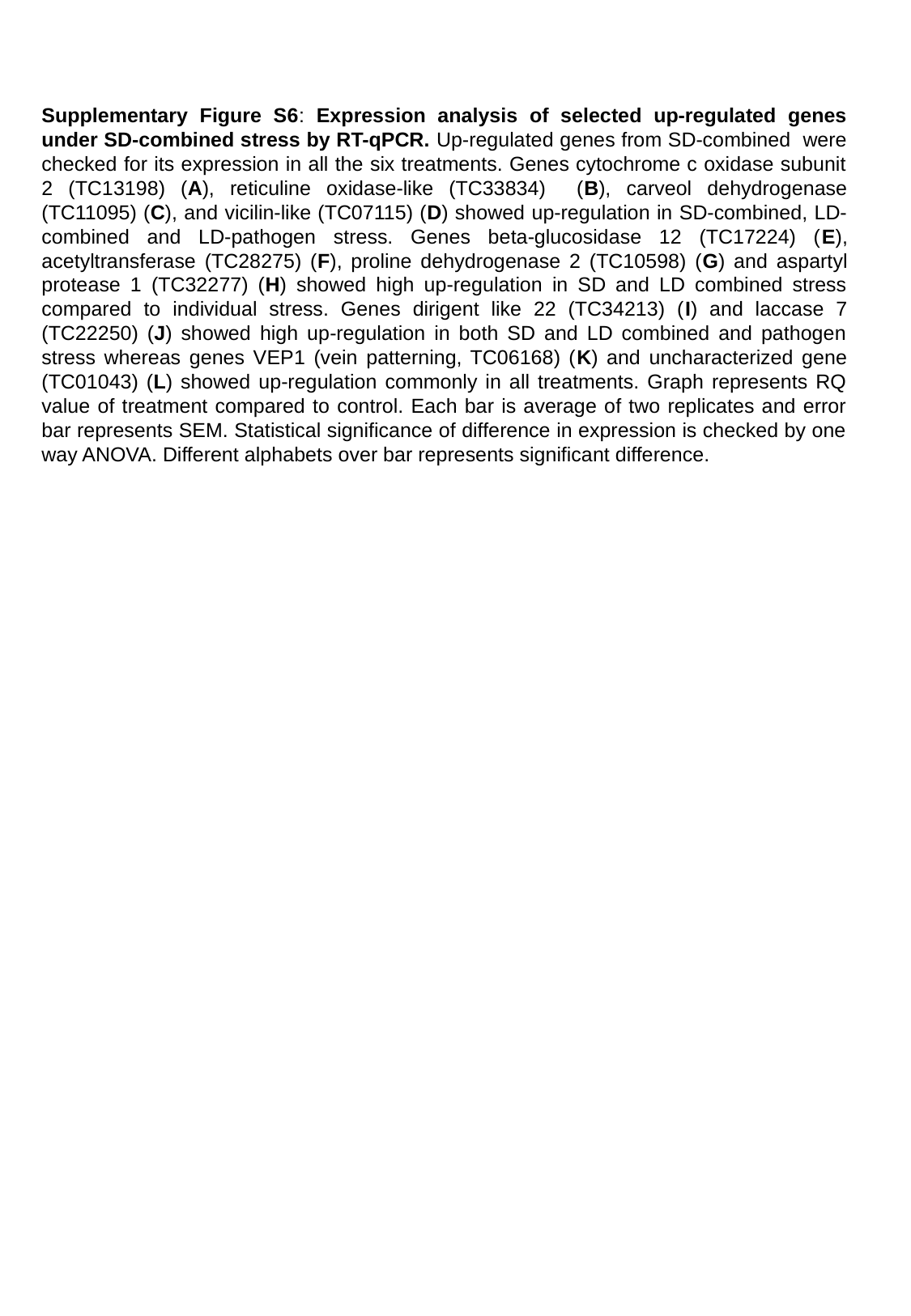

Supplementary Figure S6: Expression analysis of selected up-regulated genes under SD-combined stress by RT-qPCR. Up-regulated genes from SD-combined were checked for its expression in all the six treatments. Genes cytochrome c oxidase subunit 2 (TC13198) (A), reticuline oxidase-like (TC33834) (B), carveol dehydrogenase (TC11095) (C), and vicilin-like (TC07115) (D) showed up-regulation in SD-combined, LD-combined and LD-pathogen stress. Genes beta-glucosidase 12 (TC17224) (E), acetyltransferase (TC28275) (F), proline dehydrogenase 2 (TC10598) (G) and aspartyl protease 1 (TC32277) (H) showed high up-regulation in SD and LD combined stress compared to individual stress. Genes dirigent like 22 (TC34213) (I) and laccase 7 (TC22250) (J) showed high up-regulation in both SD and LD combined and pathogen stress whereas genes VEP1 (vein patterning, TC06168) (K) and uncharacterized gene (TC01043) (L) showed up-regulation commonly in all treatments. Graph represents RQ value of treatment compared to control. Each bar is average of two replicates and error bar represents SEM. Statistical significance of difference in expression is checked by one way ANOVA. Different alphabets over bar represents significant difference.

## Slide 12
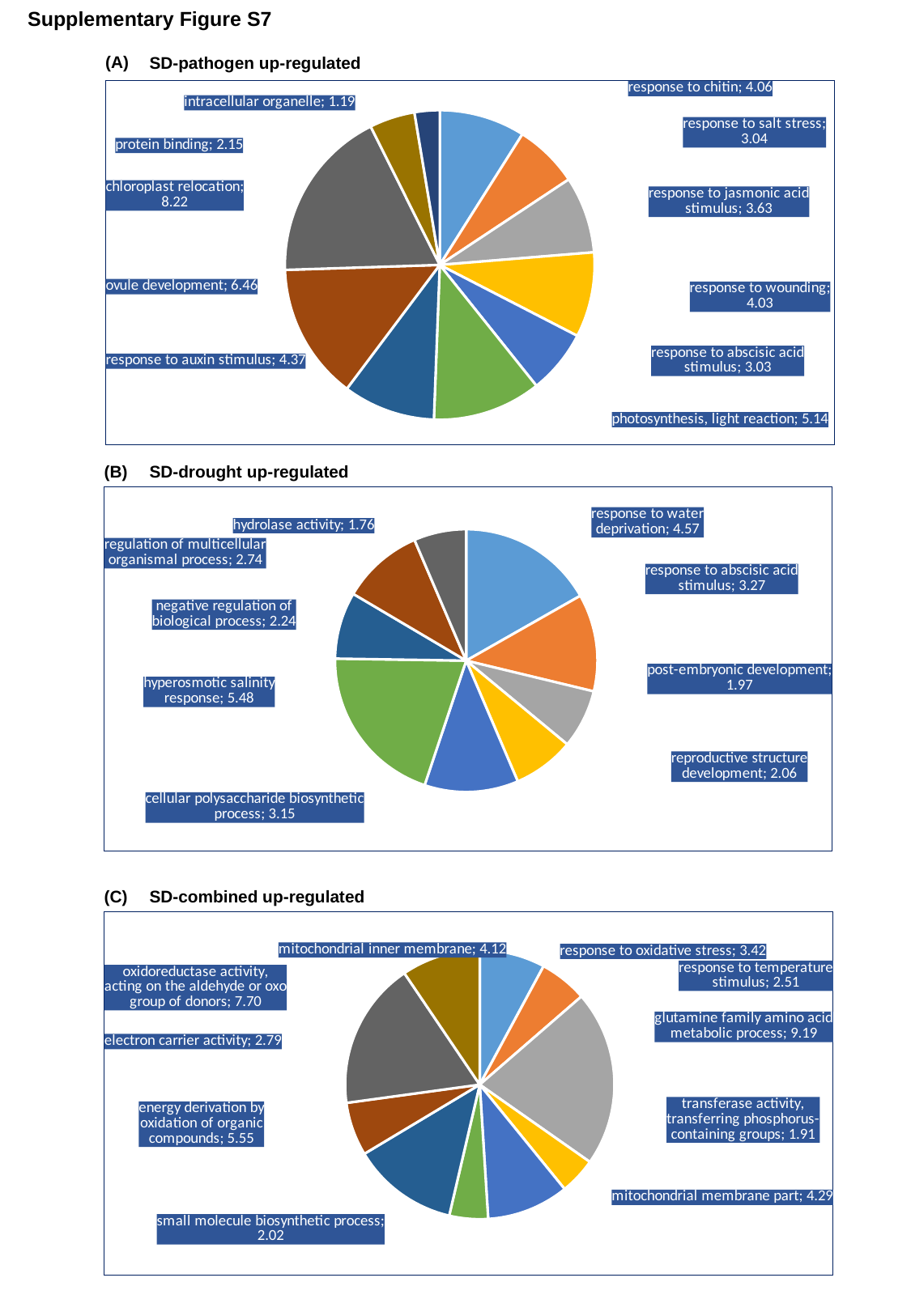

Supplementary Figure S7
(A)
SD-pathogen up-regulated
### Chart
| Category | T3 Up |
|---|---|
| response to chitin | 4.063331711644679 |
| response to salt stress | 3.0392483640144334 |
| response to jasmonic acid stimulus | 3.6319801498989586 |
| response to wounding | 4.025088589652729 |
| response to abscisic acid stimulus | 3.030159284480919 |
| photosynthesis, light reaction | 5.137125076884113 |
| response to auxin stimulus | 4.365960361166243 |
| ovule development | 6.455330756990224 |
| chloroplast relocation | 8.22433966635774 |
| protein binding | 2.147011078644589 |
| intracellular organelle | 1.187824948056312 |(B)
SD-drought up-regulated
### Chart
| Category | T2 Up |
|---|---|
| response to water deprivation | 4.5711173592032965 |
| response to abscisic acid stimulus | 3.2662755923625486 |
| post-embryonic development | 1.9699538064768867 |
| reproductive structure development | 2.06133855981417 |
| cellular polysaccharide biosynthetic process | 3.151128448511336 |
| hyperosmotic salinity response | 5.4778163580246915 |
| negative regulation of biological process | 2.243757901390645 |
| regulation of multicellular organismal process | 2.740035765747221 |
| hydrolase activity | 1.7590222076245796 |(C)
SD-combined up-regulated
### Chart
| Category | T1 UP |
|---|---|
| response to oxidative stress | 3.420848074460126 |
| response to temperature stimulus | 2.5052780569385735 |
| glutamine family amino acid metabolic process | 9.191752443840228 |
| transferase activity, transferring phosphorus-containing groups | 1.9111907911531945 |
| mitochondrial membrane part | 4.289484473792106 |
| small molecule biosynthetic process | 2.0226783930037695 |
| energy derivation by oxidation of organic compounds | 5.547492945517691 |
| electron carrier activity | 2.794292742927429 |
| oxidoreductase activity, acting on the aldehyde or oxo group of donors | 7.704851313219015 |
| mitochondrial inner membrane | 4.118226204096103 |

## Slide 13
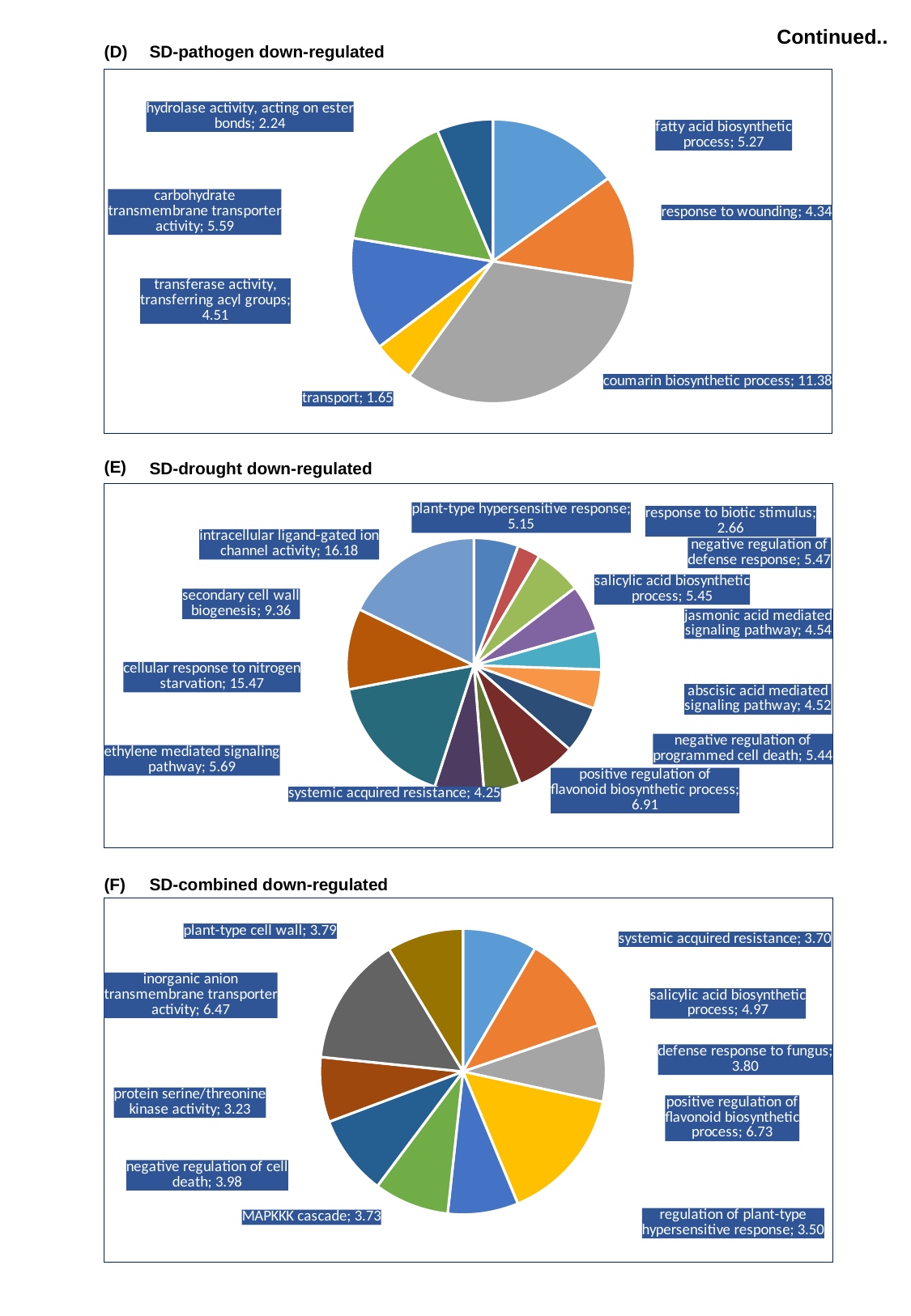

Continued..
(D)
SD-pathogen down-regulated
### Chart
| Category | T3 down |
|---|---|
| fatty acid biosynthetic process | 5.274255997028274 |
| response to wounding | 4.3387318563789155 |
| coumarin biosynthetic process | 11.382475549142216 |
| transport | 1.6496156904320172 |
| transferase activity, transferring acyl groups | 4.507460317460317 |
| carbohydrate transmembrane transporter activity | 5.587760724124361 |
| hydrolase activity, acting on ester bonds | 2.237245194141746 |(E)
SD-drought down-regulated
### Chart
| Category | T2 down |
|---|---|
| plant-type hypersensitive response | 5.1469884186776165 |
| response to biotic stimulus | 2.657816741022681 |
| negative regulation of defense response | 5.474648158858685 |
| salicylic acid biosynthetic process | 5.448453670060318 |
| jasmonic acid mediated signaling pathway | 4.542793153095505 |
| abscisic acid mediated signaling pathway | 4.518757210486534 |
| negative regulation of programmed cell death | 5.442444346159516 |
| positive regulation of flavonoid biosynthetic process | 6.909750103413874 |
| systemic acquired resistance | 4.253212713057544 |
| ethylene mediated signaling pathway | 5.693634085213032 |
| cellular response to nitrogen starvation | 15.471831753296284 |
| secondary cell wall biogenesis | 9.364529745416172 |
| intracellular ligand-gated ion channel activity | 16.175096832991567 |(F)
SD-combined down-regulated
### Chart
| Category | T1 Down |
|---|---|
| systemic acquired resistance | 3.6991388010495863 |
| salicylic acid biosynthetic process | 4.974414806750136 |
| defense response to fungus | 3.7999001996007986 |
| positive regulation of flavonoid biosynthetic process | 6.729143654438696 |
| regulation of plant-type hypersensitive response | 3.502872960278903 |
| MAPKKK cascade | 3.7308111050626023 |
| negative regulation of cell death | 3.983343657512562 |
| protein serine/threonine kinase activity | 3.2258727226398265 |
| inorganic anion transmembrane transporter activity | 6.4655018321565825 |
| plant-type cell wall | 3.7943529000393377 |

## Slide 14
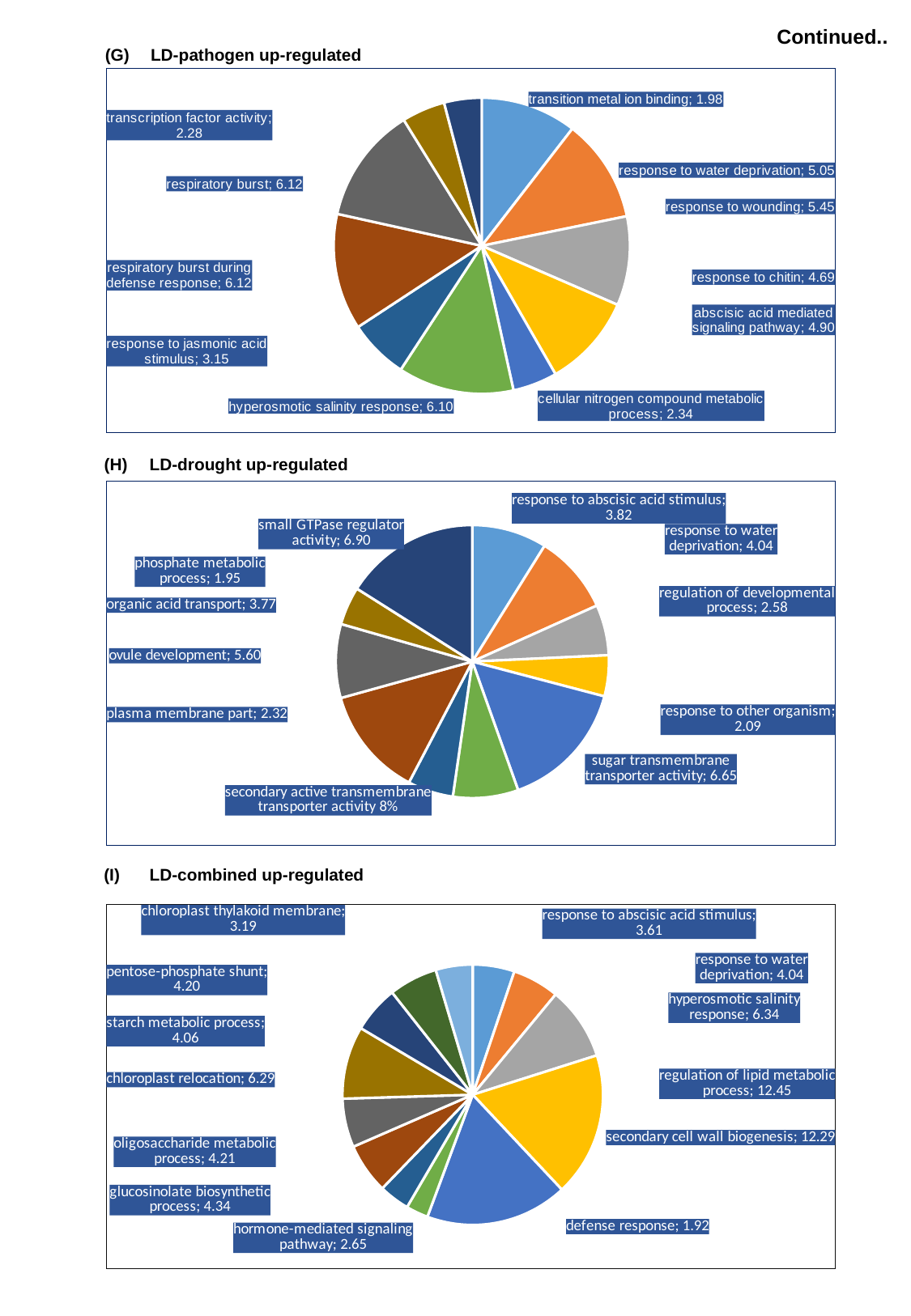

Continued..
(G)
LD-pathogen up-regulated
### Chart
| Category | T6 Up |
|---|---|
| response to water deprivation | 5.045453595317726 |
| response to wounding | 5.4469948849104854 |
| response to chitin | 4.692264793968811 |
| abscisic acid mediated signaling pathway | 4.899413388543824 |
| cellular nitrogen compound metabolic process | 2.3380157699737167 |
| hyperosmotic salinity response | 6.097047772410091 |
| response to jasmonic acid stimulus | 3.1456106341733587 |
| respiratory burst during defense response | 6.1222421846927775 |
| respiratory burst | 6.1222421846927775 |
| transcription factor activity | 2.275518275345086 |
| transition metal ion binding | 1.9750164476775878 |(H)
LD-drought up-regulated
### Chart
| Category | T5 Up |
|---|---|
| response to abscisic acid stimulus | 3.8239324008146918 |
| response to water deprivation | 4.043394867327795 |
| regulation of developmental process | 2.5791155440328946 |
| response to other organism | 2.088900440628011 |
| sugar transmembrane transporter activity | 6.6517143442742945 |
| secondary active transmembrane transporter activity | 3.3314562582855265 |
| plasma membrane part | 2.3226349969737123 |
| ovule development | 5.600617973834725 |
| organic acid transport | 3.766044150802358 |
| phosphate metabolic process | 1.9524327033758653 |
| small GTPase regulator activity | 6.90308727007536 |(I)
LD-combined up-regulated
### Chart
| Category | T4 Up |
|---|---|
| response to abscisic acid stimulus | 3.610094075769133 |
| response to water deprivation | 4.041830086032388 |
| hyperosmotic salinity response | 6.34273473034438 |
| regulation of lipid metabolic process | 12.454824561403507 |
| secondary cell wall biogenesis | 12.290945290858726 |
| defense response | 1.9213431719043526 |
| hormone-mediated signaling pathway | 2.6466502192982455 |
| glucosinolate biosynthetic process | 4.344706242350061 |
| oligosaccharide metabolic process | 4.2111599439171705 |
| chloroplast relocation | 6.287291244939271 |
| starch metabolic process | 4.061355835240275 |
| pentose-phosphate shunt | 4.203503289473684 |
| chloroplast thylakoid membrane | 3.191065299117359 |

## Slide 15
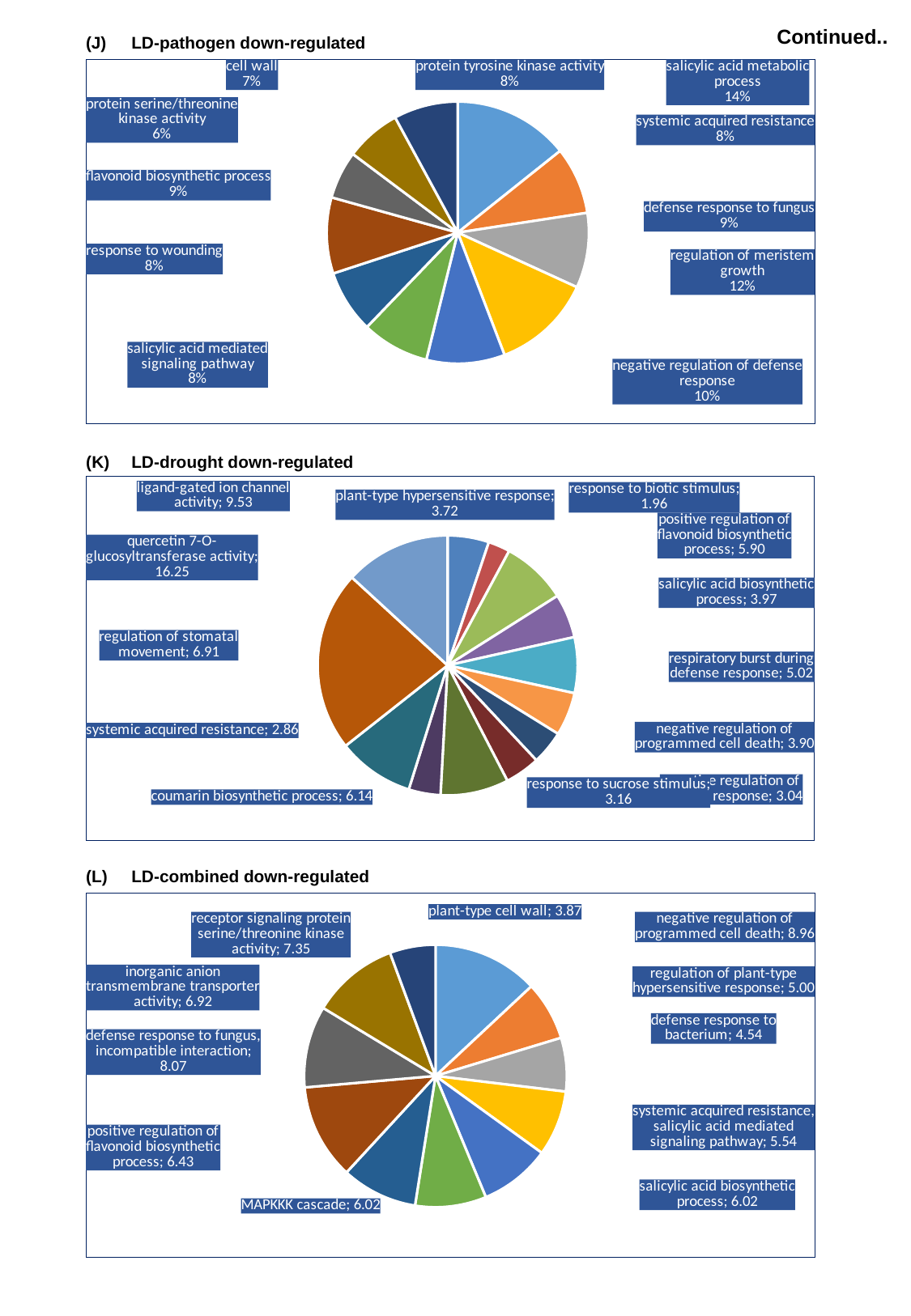

Continued..
(J)
LD-pathogen down-regulated
### Chart
| Category | T6 Down |
|---|---|
| salicylic acid metabolic process | 6.316761205650095 |
| systemic acquired resistance | 3.67649697137837 |
| defense response to fungus | 4.100353765071113 |
| regulation of meristem growth | 5.467138353428152 |
| negative regulation of defense response | 4.28058909540391 |
| salicylic acid mediated signaling pathway | 3.6832690697701844 |
| response to wounding | 3.4370612442507866 |
| flavonoid biosynthetic process | 4.155025148605397 |
| protein serine/threonine kinase activity | 2.610703966377725 |
| cell wall | 3.025503748984512 |
| protein tyrosine kinase activity | 3.509311780916719 |(K)
LD-drought down-regulated
### Chart
| Category | T5 down |
|---|---|
| plant-type hypersensitive response | 3.7198783197647907 |
| response to biotic stimulus | 1.96492288246813 |
| positive regulation of flavonoid biosynthetic process | 5.9001737750746095 |
| salicylic acid biosynthetic process | 3.9651015582819804 |
| respiratory burst during defense response | 5.022461973823842 |
| negative regulation of programmed cell death | 3.8997940032043945 |
| negative regulation of defense response | 3.035553940223201 |
| response to sucrose stimulus | 3.156976097832129 |
| coumarin biosynthetic process | 6.138564634673584 |
| systemic acquired resistance | 2.861402638473344 |
| regulation of stomatal movement | 6.905885214007782 |
| quercetin 7-O-glucosyltransferase activity | 16.24914168001831 |
| ligand-gated ion channel activity | 9.525358915872804 |(L)
LD-combined down-regulated
### Chart
| Category | T4 down |
|---|---|
| negative regulation of programmed cell death | 8.955587549705196 |
| regulation of plant-type hypersensitive response | 4.995733825922505 |
| defense response to bacterium | 4.536100954882682 |
| systemic acquired resistance, salicylic acid mediated signaling pathway | 5.538099350848355 |
| salicylic acid biosynthetic process | 6.017588472133927 |
| MAPKKK cascade | 6.017588472133927 |
| positive regulation of flavonoid biosynthetic process | 6.426550795482835 |
| defense response to fungus, incompatible interaction | 8.072374779691854 |
| inorganic anion transmembrane transporter activity | 6.9157360052882435 |
| receptor signaling protein serine/threonine kinase activity | 7.354830354830355 |
| plant-type cell wall | 3.865312303268508 |

## Slide 16
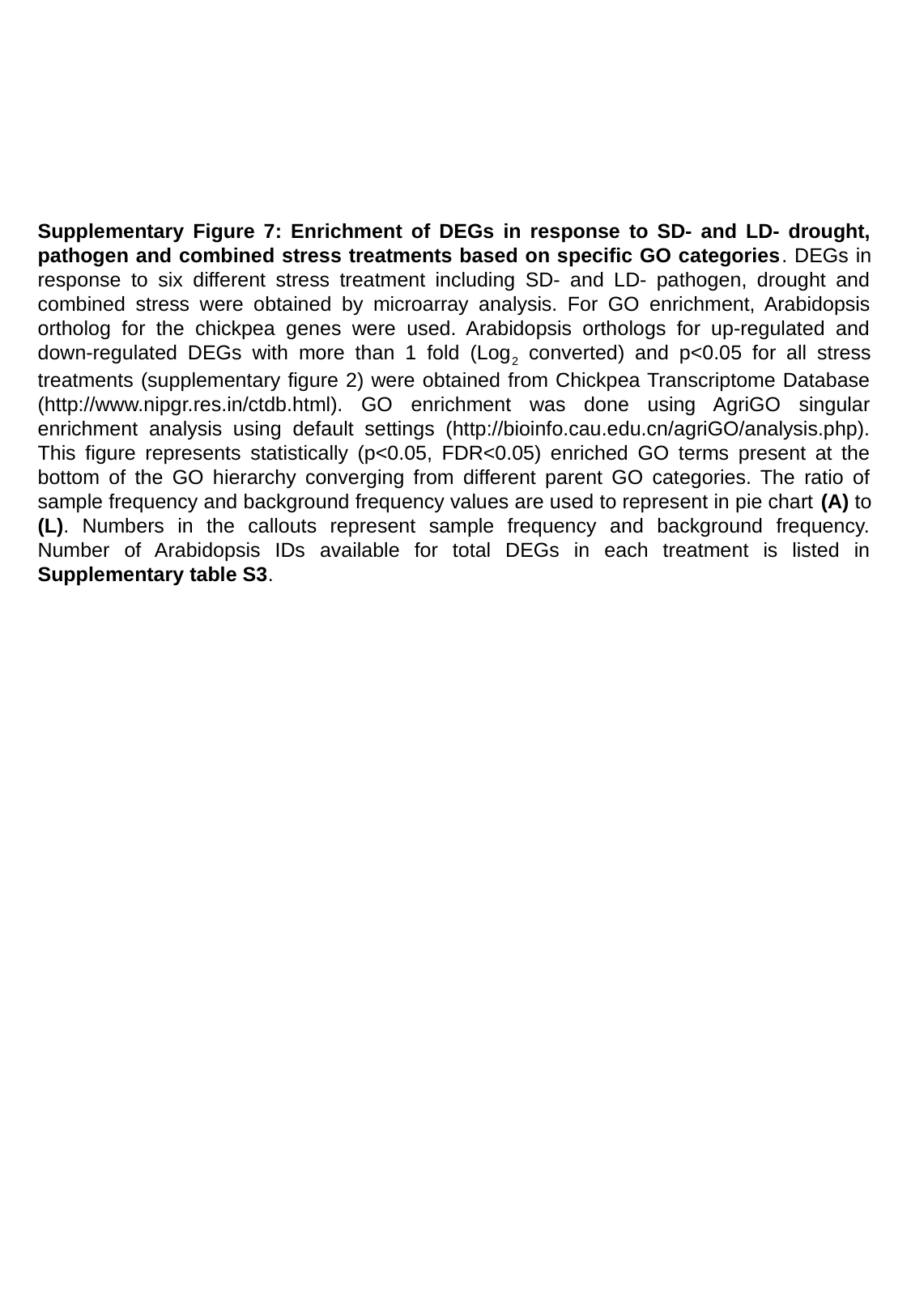

Supplementary Figure 7: Enrichment of DEGs in response to SD- and LD- drought, pathogen and combined stress treatments based on specific GO categories. DEGs in response to six different stress treatment including SD- and LD- pathogen, drought and combined stress were obtained by microarray analysis. For GO enrichment, Arabidopsis ortholog for the chickpea genes were used. Arabidopsis orthologs for up-regulated and down-regulated DEGs with more than 1 fold (Log2 converted) and p<0.05 for all stress treatments (supplementary figure 2) were obtained from Chickpea Transcriptome Database (http://www.nipgr.res.in/ctdb.html). GO enrichment was done using AgriGO singular enrichment analysis using default settings (http://bioinfo.cau.edu.cn/agriGO/analysis.php). This figure represents statistically (p<0.05, FDR<0.05) enriched GO terms present at the bottom of the GO hierarchy converging from different parent GO categories. The ratio of sample frequency and background frequency values are used to represent in pie chart (A) to (L). Numbers in the callouts represent sample frequency and background frequency. Number of Arabidopsis IDs available for total DEGs in each treatment is listed in Supplementary table S3.

## Slide 17
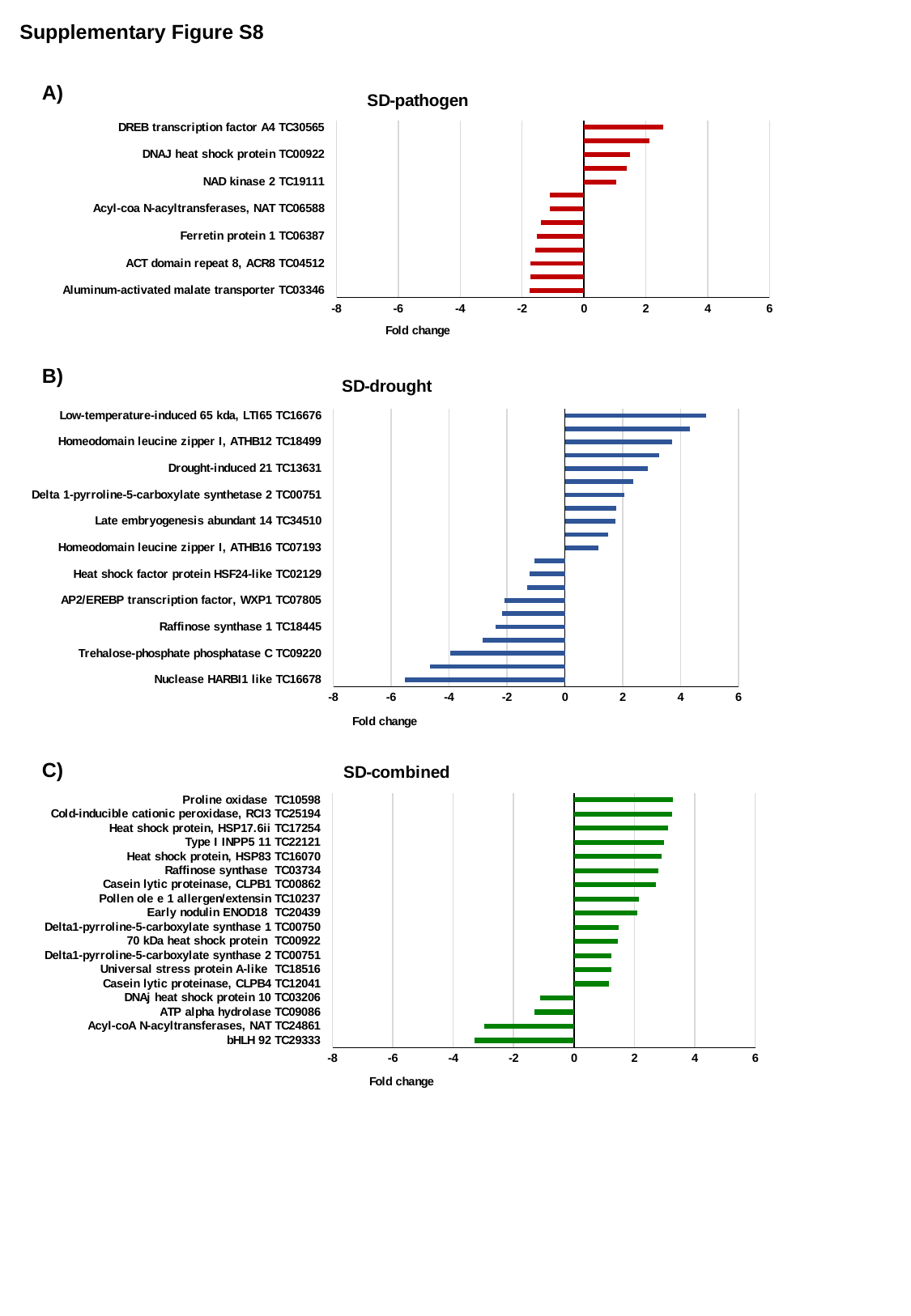

Supplementary Figure S8
A)
### Chart: SD-pathogen
| Category | |
|---|---|
| Aluminum-activated malate transporter TC03346 | -1.7520467 |
| Responsive to dehydration 22, RD22 TC05736 | -1.7281538 |
| ACT domain repeat 8, ACR8 TC04512 | -1.7252171 |
| Ferretin protein 3 TC11561 | -1.5598043999999998 |
| Ferretin protein 1 TC06387 | -1.5197113999999998 |
| SPX domain gene 2 TC08685 | -1.3853585 |
| Acyl-coa N-acyltransferases, NAT TC06588 | -1.1119955999999998 |
| Drought-responsive family protein TC18323 | -1.0907158 |
| NAD kinase 2 TC19111 | 1.0462296999999998 |
| Fatty acid desaturase 2 TC30715 | 1.3928894 |
| DNAJ heat shock protein TC00922 | 1.5016408999999997 |
| Pollen Ole e 1 allergen/extensin TC10237 | 2.1295252 |
| DREB transcription factor A4 TC30565 | 2.5717833 |B)
### Chart: SD-drought
| Category | |
|---|---|
| Nuclease HARBI1 like TC16678 | -5.513513599999999 |
| Chaperone protein dnaJ 20 TC34623 | -4.6637034 |
| Trehalose-phosphate phosphatase C TC09220 | -3.9482189999999995 |
| Alpha-dioxygenase TC02051 | -2.8351449999999994 |
| Raffinose synthase 1 TC18445 | -2.3881927 |
| DREB A4 TC09635 | -2.166584 |
| AP2/EREBP transcription factor, WXP1 TC07805 | -2.0954527999999994 |
| Trehalose-phosphate phosphatase A-like TC21306 | -1.3034333999999999 |
| Heat shock factor protein HSF24-like TC02129 | -1.2169175 |
| AP2/EREBP transcription factor, WXP2 TC16262 | -1.0526814 |
| Homeodomain leucine zipper I, ATHB16 TC07193 | 1.1565151000000002 |
| Delta 1-pyrroline-5-carboxylate synthetase 1 TC00750 | 1.4857606999999995 |
| Late embryogenesis abundant 14 TC34510 | 1.7449082999999999 |
| Heat shock 70 kDa protein 8 TC30008 | 1.7619045999999996 |
| Delta 1-pyrroline-5-carboxylate synthetase 2 TC00751 | 2.0481892 |
| Rafinose synthase 2 TC03734 | 2.3508052999999993 |
| Drought-induced 21 TC13631 | 2.872075999999999 |
| Verbascose synthase TC07956 | 3.2727106 |
| Homeodomain leucine zipper I, ATHB12 TC18499 | 3.7063298000000002 |
| Choline kinase TC19235 | 4.318993599999999 |
| Low-temperature-induced 65 kda, LTI65 TC16676 | 4.893052 |
### Chart: SD-combined
| Category | |
|---|---|
| bHLH 92 TC29333 | -3.3013033999999997 |
| Acyl-coA N-acyltransferases, NAT TC24861 | -2.9589955999999997 |
| ATP alpha hydrolase TC09086 | -1.3039929999999997 |
| DNAj heat shock protein 10 TC03206 | -1.1212694999999997 |
| Casein lytic proteinase, CLPB4 TC12041 | 1.1513271 |
| Universal stress protein A-like TC18516 | 1.2257396999999997 |
| Delta1-pyrroline-5-carboxylate synthase 2 TC00751 | 1.2465111999999998 |
| 70 kDa heat shock protein  TC00922 | 1.4481373 |
| Delta1-pyrroline-5-carboxylate synthase 1 TC00750 | 1.4664294999999998 |
| Early nodulin ENOD18  TC20439 | 2.0839740000000004 |
| Pollen ole e 1 allergen/extensin TC10237 | 2.1594984999999998 |
| Casein lytic proteinase, CLPB1 TC00862 | 2.7004201 |
| Raffinose synthase TC03734 | 2.7958592999999996 |
| Heat shock protein, HSP83 TC16070 | 2.8921417999999997 |
| Type I INPP5 11 TC22121 | 2.9790689999999995 |
| Heat shock protein, HSP17.6ii TC17254 | 3.10442 |
| Cold-inducible cationic peroxidase, RCI3 TC25194 | 3.2512502999999997 |
| Proline oxidase TC10598 | 3.2648737 |C)

## Slide 18
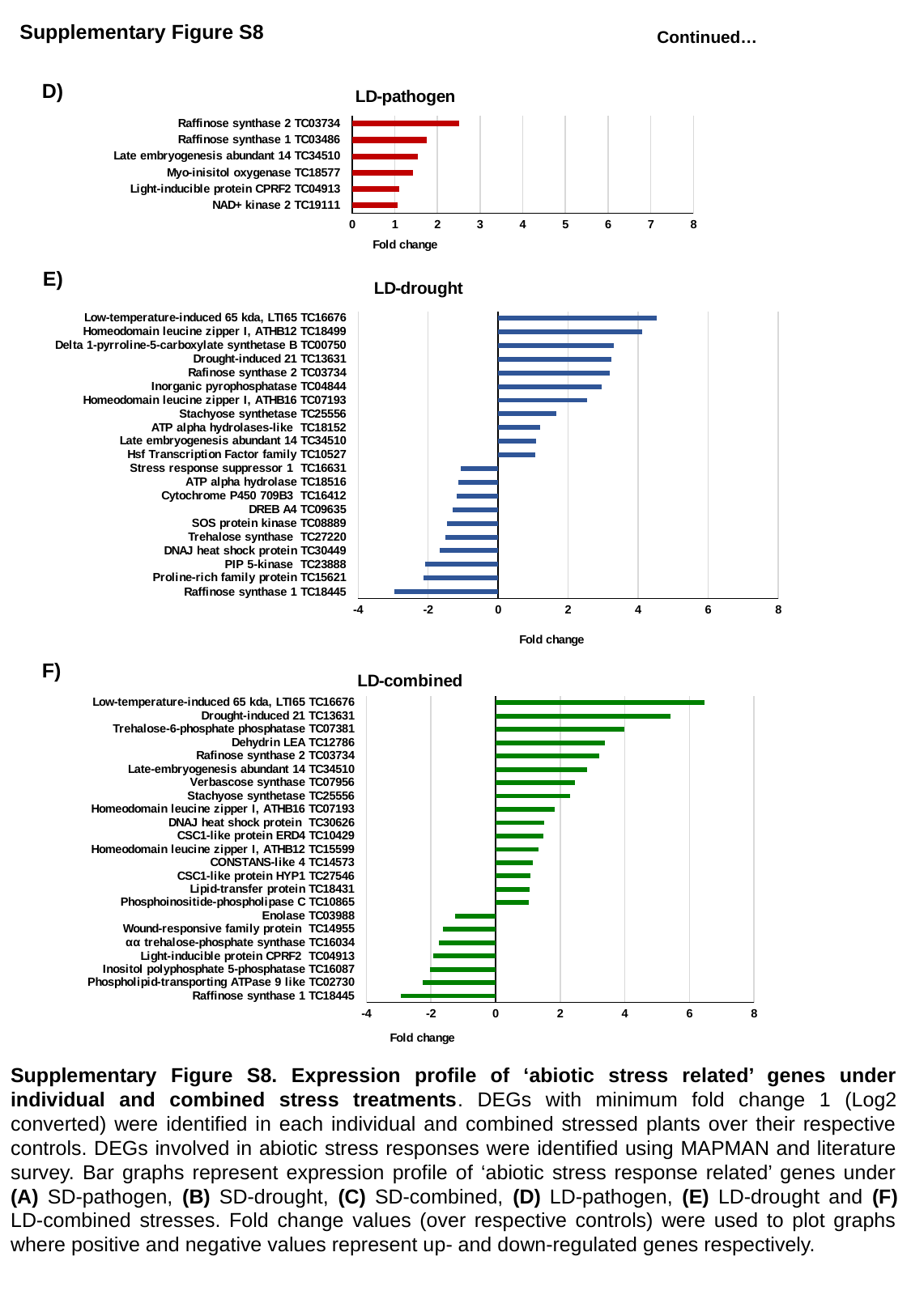

Supplementary Figure S8
Continued…
D)
### Chart: LD-pathogen
| Category | |
|---|---|
| NAD+ kinase 2 TC19111 | 1.0565011999999998 |
| Light-inducible protein CPRF2 TC04913 | 1.1062083 |
| Myo-inisitol oxygenase TC18577 | 1.4221528 |
| Late embryogenesis abundant 14 TC34510 | 1.5482466 |
| Raffinose synthase 1 TC03486 | 1.7453162999999998 |
| Raffinose synthase 2 TC03734 | 2.5068754999999996 |E)
### Chart: LD-drought
| Category | |
|---|---|
| Raffinose synthase 1 TC18445 | -2.9694459999999996 |
| Proline-rich family protein TC15621 | -2.130385 |
| PIP 5-kinase TC23888 | -2.0969585999999993 |
| DNAJ heat shock protein TC30449 | -1.6762520000000003 |
| Trehalose synthase TC27220 | -1.4978295999999995 |
| SOS protein kinase TC08889 | -1.4689446999999998 |
| DREB A4 TC09635 | -1.2911356999999999 |
| Cytochrome P450 709B3 TC16412 | -1.1839559000000002 |
| ATP alpha hydrolase TC18516 | -1.1451356 |
| Stress response suppressor 1 TC16631 | -1.0614101999999999 |
| Hsf Transcription Factor family TC10527 | 1.0580365999999999 |
| Late embryogenesis abundant 14 TC34510 | 1.0827395999999998 |
| ATP alpha hydrolases-like TC18152 | 1.1904058000000002 |
| Stachyose synthetase TC25556 | 1.6658225 |
| Homeodomain leucine zipper I, ATHB16 TC07193 | 2.5498451999999996 |
| Inorganic pyrophosphatase TC04844 | 2.9519640999999996 |
| Rafinose synthase 2 TC03734 | 3.1816478 |
| Drought-induced 21 TC13631 | 3.2266589999999997 |
| Delta 1-pyrroline-5-carboxylate synthetase B TC00750 | 3.2915418 |
| Homeodomain leucine zipper I, ATHB12 TC18499 | 4.114859599999999 |
| Low-temperature-induced 65 kda, LTI65 TC16676 | 4.5349189999999995 |F)
### Chart: LD-combined
| Category | |
|---|---|
| Raffinose synthase 1 TC18445 | -2.9260259999999993 |
| Phospholipid-transporting ATPase 9 like TC02730 | -2.2637932000000003 |
| Inositol polyphosphate 5-phosphatase TC16087 | -2.0268475999999995 |
| Light-inducible protein CPRF2  TC04913 | -1.9235796999999997 |
| αα trehalose-phosphate synthase TC16034 | -1.7504601 |
| Wound-responsive family protein TC14955 | -1.6317612999999997 |
| Enolase TC03988 | -1.2574395999999997 |
| Phosphoinositide-phospholipase C TC10865 | 1.0275430999999997 |
| Lipid-transfer protein TC18431 | 1.0487343999999998 |
| CSC1-like protein HYP1 TC27546 | 1.0719856999999997 |
| CONSTANS-like 4 TC14573 | 1.1627197 |
| Homeodomain leucine zipper I, ATHB12 TC15599 | 1.334033 |
| CSC1-like protein ERD4 TC10429 | 1.4882551 |
| DNAJ heat shock protein TC30626 | 1.4931679999999998 |
| Homeodomain leucine zipper I, ATHB16 TC07193 | 1.8266462999999997 |
| Stachyose synthetase TC25556 | 2.3046416999999995 |
| Verbascose synthase TC07956 | 2.4552416999999997 |
| Late-embryogenesis abundant 14 TC34510 | 2.8379946 |
| Rafinose synthase 2 TC03734 | 3.2047162000000005 |
| Dehydrin LEA TC12786 | 3.3800596999999994 |
| Trehalose-6-phosphate phosphatase TC07381 | 3.9860534999999997 |
| Drought-induced 21 TC13631 | 5.409620300000001 |
| Low-temperature-induced 65 kda, LTI65 TC16676 | 6.466858 |Supplementary Figure S8. Expression profile of ‘abiotic stress related’ genes under individual and combined stress treatments. DEGs with minimum fold change 1 (Log2 converted) were identified in each individual and combined stressed plants over their respective controls. DEGs involved in abiotic stress responses were identified using MAPMAN and literature survey. Bar graphs represent expression profile of ‘abiotic stress response related’ genes under (A) SD-pathogen, (B) SD-drought, (C) SD-combined, (D) LD-pathogen, (E) LD-drought and (F) LD-combined stresses. Fold change values (over respective controls) were used to plot graphs where positive and negative values represent up- and down-regulated genes respectively.

## Slide 19
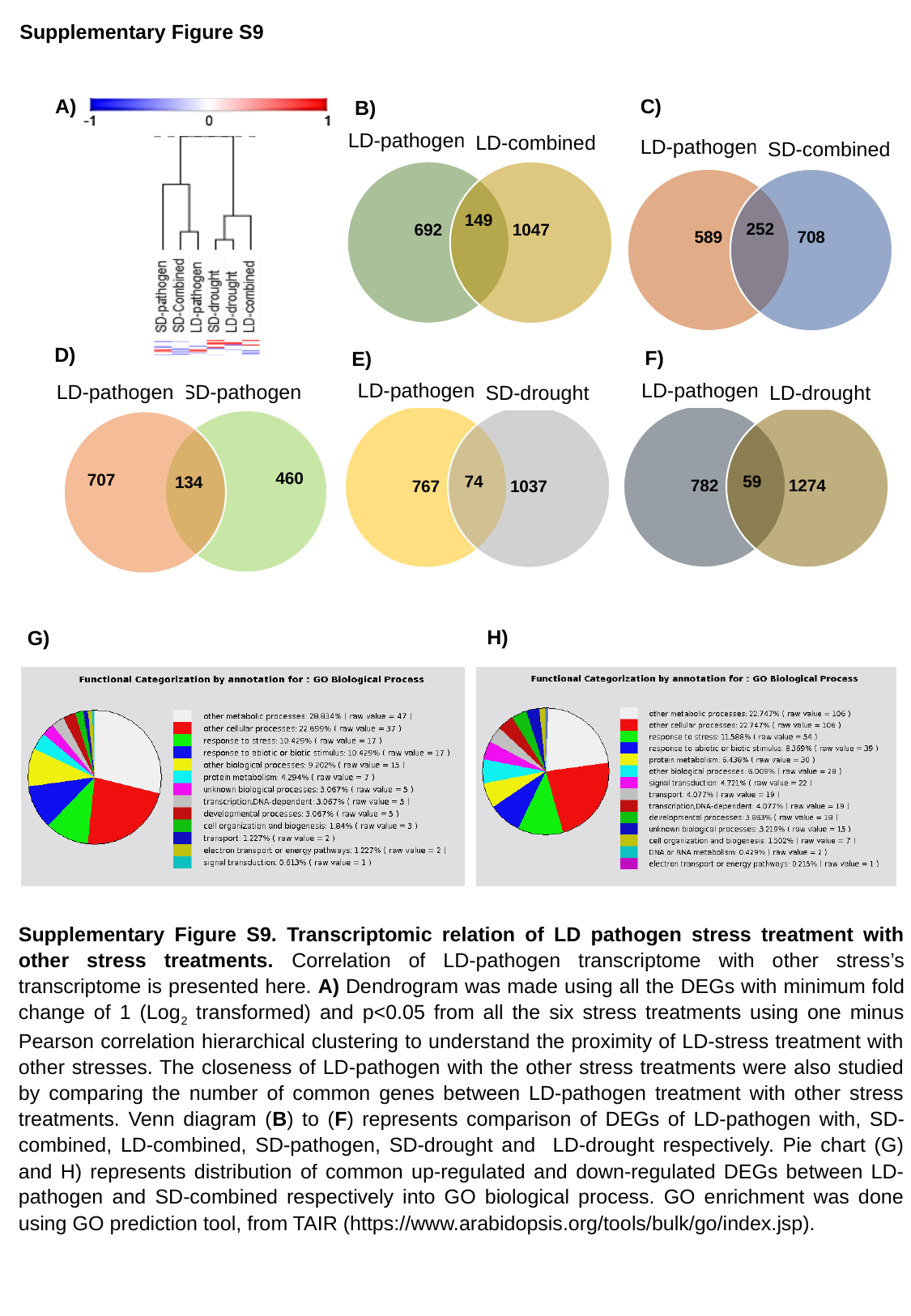

Supplementary Figure S9
 149
LD-pathogen
LD-combined
A)
C)
B)
 252
LD-pathogen
SD-combined
D)
F)
 134
LD-pathogen
SD-pathogen
E)
 59
LD-pathogen
LD-drought
 74
LD-pathogen
SD-drought
H)
G)
Supplementary Figure S9. Transcriptomic relation of LD pathogen stress treatment with other stress treatments. Correlation of LD-pathogen transcriptome with other stress’s transcriptome is presented here. A) Dendrogram was made using all the DEGs with minimum fold change of 1 (Log2 transformed) and p<0.05 from all the six stress treatments using one minus Pearson correlation hierarchical clustering to understand the proximity of LD-stress treatment with other stresses. The closeness of LD-pathogen with the other stress treatments were also studied by comparing the number of common genes between LD-pathogen treatment with other stress treatments. Venn diagram (B) to (F) represents comparison of DEGs of LD-pathogen with, SD-combined, LD-combined, SD-pathogen, SD-drought and LD-drought respectively. Pie chart (G) and H) represents distribution of common up-regulated and down-regulated DEGs between LD-pathogen and SD-combined respectively into GO biological process. GO enrichment was done using GO prediction tool, from TAIR (https://www.arabidopsis.org/tools/bulk/go/index.jsp).

## Slide 20
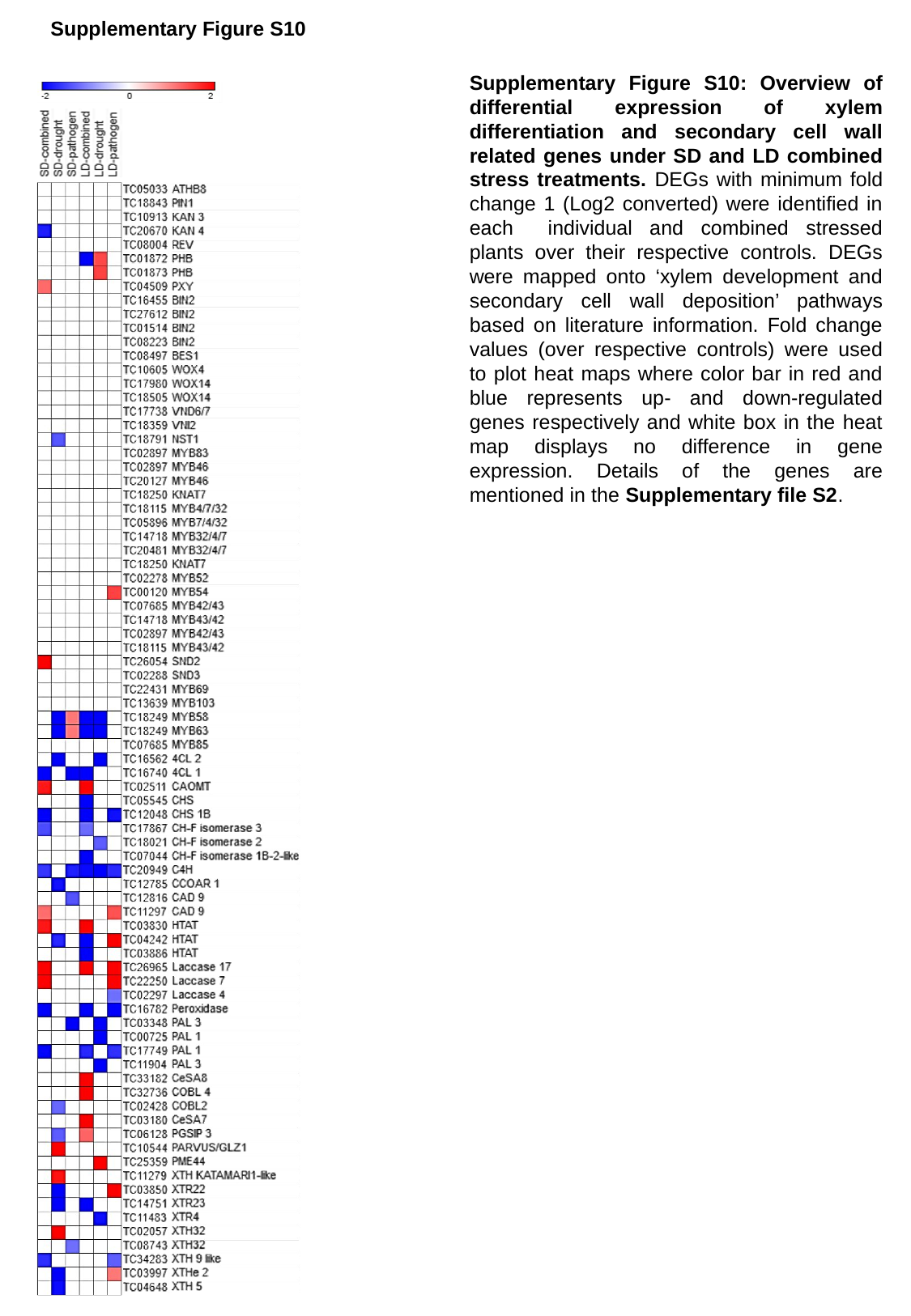

Supplementary Figure S10
Supplementary Figure S10: Overview of differential expression of xylem differentiation and secondary cell wall related genes under SD and LD combined stress treatments. DEGs with minimum fold change 1 (Log2 converted) were identified in each individual and combined stressed plants over their respective controls. DEGs were mapped onto ‘xylem development and secondary cell wall deposition’ pathways based on literature information. Fold change values (over respective controls) were used to plot heat maps where color bar in red and blue represents up- and down-regulated genes respectively and white box in the heat map displays no difference in gene expression. Details of the genes are mentioned in the Supplementary file S2.

## Slide 21
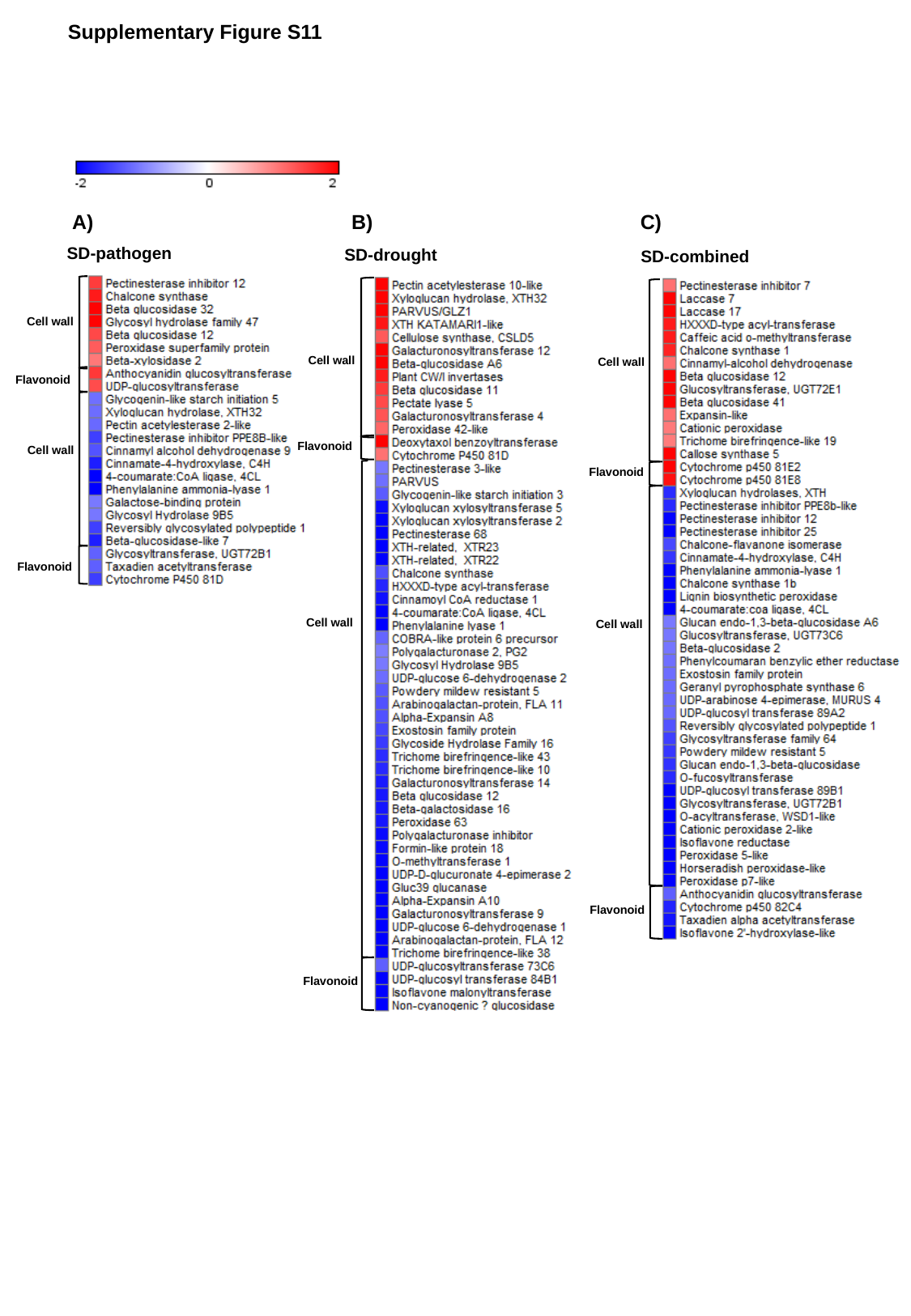

Supplementary Figure S11
C)
A)
B)
SD-pathogen
SD-drought
SD-combined
Cell wall
Flavonoid
Cell wall
Flavonoid
Cell wall
Flavonoid
Cell wall
Flavonoid
Cell wall
Flavonoid
Cell wall
Flavonoid

## Slide 22
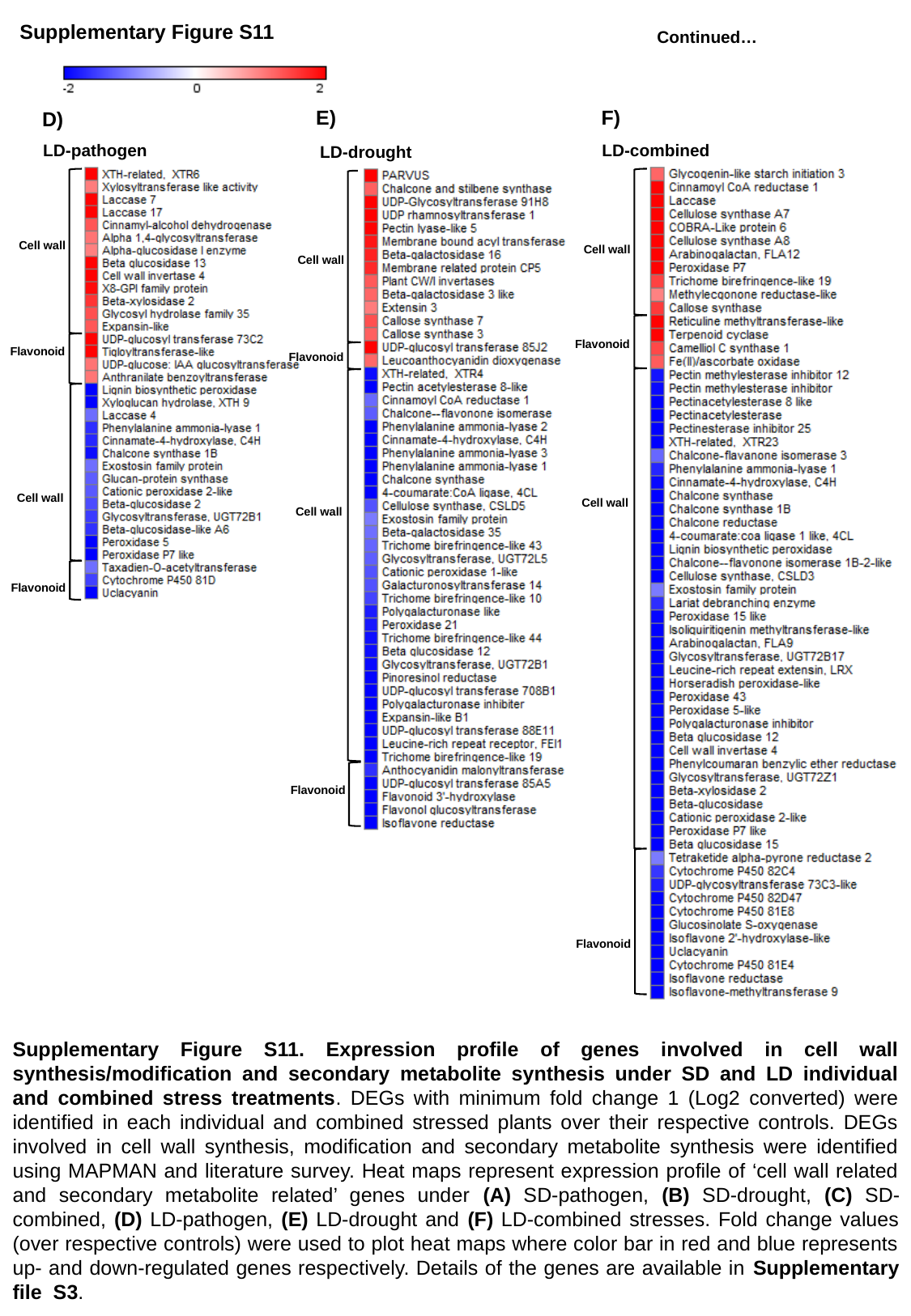

Supplementary Figure S11
Continued…
F)
E)
D)
LD-combined
LD-pathogen
LD-drought
Cell wall
Flavonoid
Cell wall
Flavonoid
Cell wall
Flavonoid
Cell wall
Flavonoid
Cell wall
Flavonoid
Cell wall
Flavonoid
Supplementary Figure S11. Expression profile of genes involved in cell wall synthesis/modification and secondary metabolite synthesis under SD and LD individual and combined stress treatments. DEGs with minimum fold change 1 (Log2 converted) were identified in each individual and combined stressed plants over their respective controls. DEGs involved in cell wall synthesis, modification and secondary metabolite synthesis were identified using MAPMAN and literature survey. Heat maps represent expression profile of ‘cell wall related and secondary metabolite related’ genes under (A) SD-pathogen, (B) SD-drought, (C) SD-combined, (D) LD-pathogen, (E) LD-drought and (F) LD-combined stresses. Fold change values (over respective controls) were used to plot heat maps where color bar in red and blue represents up- and down-regulated genes respectively. Details of the genes are available in Supplementary file S3.

## Slide 23
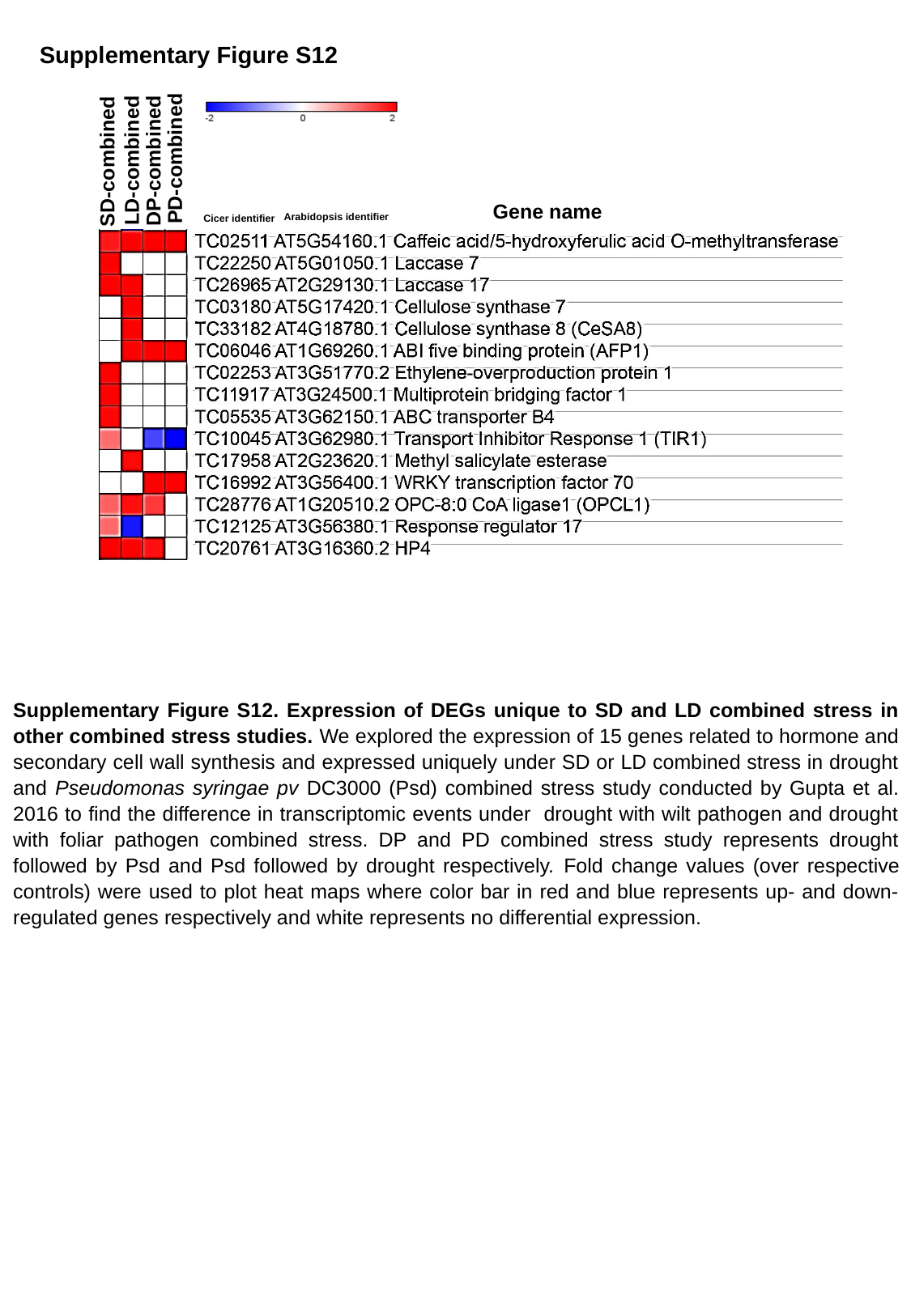

Supplementary Figure S12
PD-combined
LD-combined
DP-combined
SD-combined
Gene name
Arabidopsis identifier
Cicer identifier
Supplementary Figure S12. Expression of DEGs unique to SD and LD combined stress in other combined stress studies. We explored the expression of 15 genes related to hormone and secondary cell wall synthesis and expressed uniquely under SD or LD combined stress in drought and Pseudomonas syringae pv DC3000 (Psd) combined stress study conducted by Gupta et al. 2016 to find the difference in transcriptomic events under drought with wilt pathogen and drought with foliar pathogen combined stress. DP and PD combined stress study represents drought followed by Psd and Psd followed by drought respectively. Fold change values (over respective controls) were used to plot heat maps where color bar in red and blue represents up- and down-regulated genes respectively and white represents no differential expression.

## Slide 24
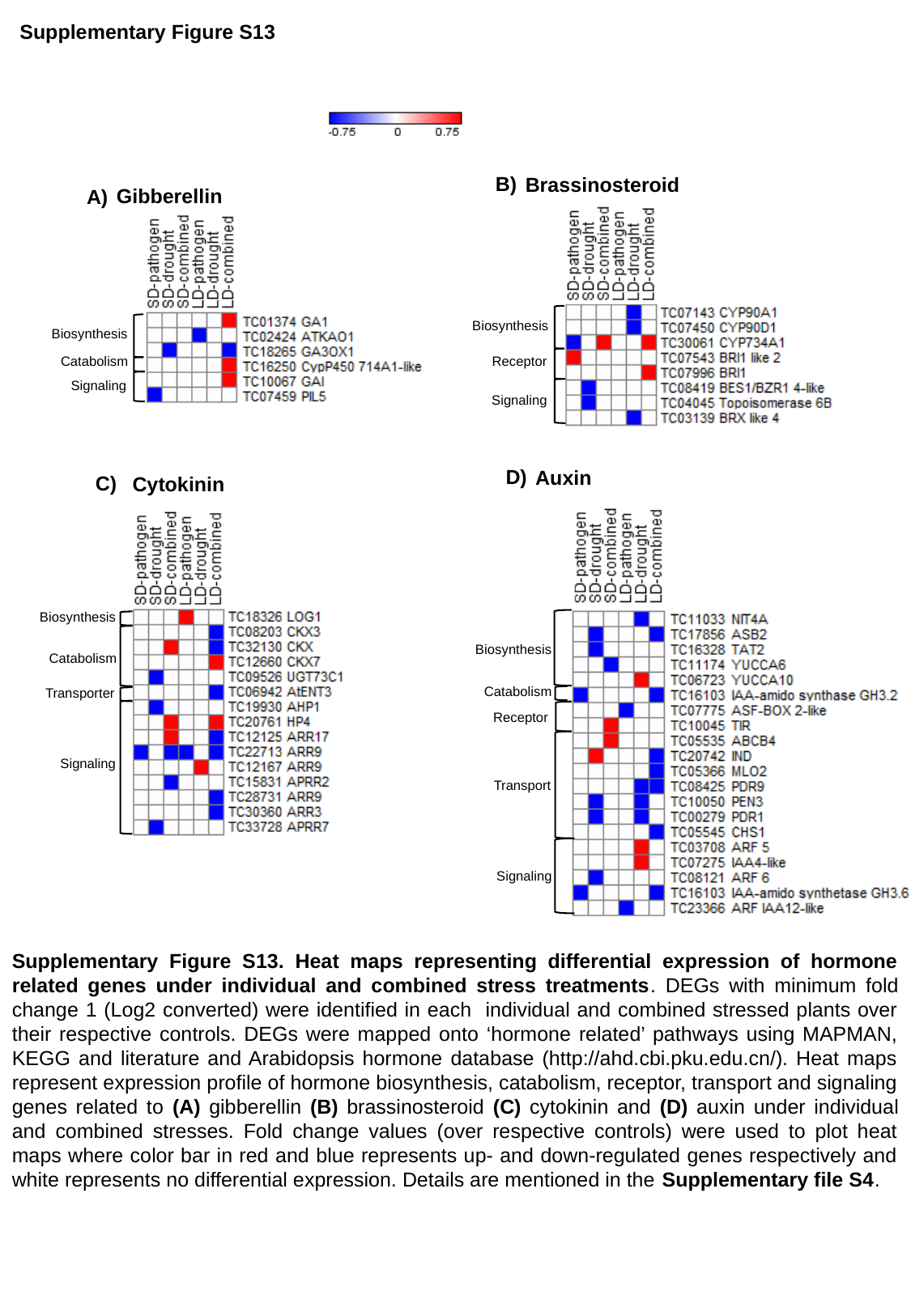

Supplementary Figure S13
B)
Brassinosteroid
Gibberellin
A)
Biosynthesis
Receptor
Signaling
Biosynthesis
Catabolism
Signaling
D)
Auxin
C)
Cytokinin
Biosynthesis
Catabolism
Transporter
Signaling
Biosynthesis
Catabolism
Receptor
Transport
Signaling
Supplementary Figure S13. Heat maps representing differential expression of hormone related genes under individual and combined stress treatments. DEGs with minimum fold change 1 (Log2 converted) were identified in each individual and combined stressed plants over their respective controls. DEGs were mapped onto ‘hormone related’ pathways using MAPMAN, KEGG and literature and Arabidopsis hormone database (http://ahd.cbi.pku.edu.cn/). Heat maps represent expression profile of hormone biosynthesis, catabolism, receptor, transport and signaling genes related to (A) gibberellin (B) brassinosteroid (C) cytokinin and (D) auxin under individual and combined stresses. Fold change values (over respective controls) were used to plot heat maps where color bar in red and blue represents up- and down-regulated genes respectively and white represents no differential expression. Details are mentioned in the Supplementary file S4.
